# Supplementary material for: Conformational dynamics of the human serotonin transporter during substrate and drug binding
Source: Nat Commun. 2019 Apr 11;10:1687. doi: 10.1038/s41467-019-09675-z (PMC6459873; doi:10.1038/s41467-019-09675-z)
Supplement: Supplementary file 1 — Supplementary Information [file 41467_2019_9675_MOESM1_ESM.pdf]

Conformational Dynamics of the Human Serotonin Transporter during Substrate and Drug Binding

Möller et al.

Supplementary information

Supplementary Results

Evaluating the purity and stability of hSERT

The purity of the hSERT was evaluated by SDS-PAGE and western blot, and saturation binding using scintillation proximity assay was used to evaluate the activity (see Supplementary Figure 1a). By SDS-PAGE, the transporter was determined to be around 20-25% pure. In a western blot using an antibody specific for the C-terminal of hSERT, degradation products or products from proteolysis could not be observed (see Supplementary Figure 1b). The dissociation constant of imipramine was determined to be 7.5 +/- 0.7 nM (see Supplementary Figure 1c).

Size exclusion chromatography (SEC) was used to evaluate the oligomeric state of purified hSERT. SEC showed three main peaks, one corresponding to the void volume of the column, one corresponding roughly to monomeric SERT, and finally one corresponding to the elution of imidazole. SDS-PAGE from the two first peaks showed that the majority of SERT was in the peak corresponding to monomeric SERT. We note, however, that the SERT is glycosylated, adding a level of native heterogeneity and the sample was solubilized in detergent that results in micelles of varied sizes and thus SEC is in fact not the ideal tool to assess for partial unfolding or aggregation of SERT. In accordance, SDS-PAGE revealed that smaller amounts of SERT were also detected in fractions collected between the main SERT peak up to and including the void volume peak. To verify that the dynamics of hSERT from the main SERT peak and hSERT prior to SEC-purification was similar, SEC fractions of the main SERT peak were collected and up-concentrated and subjected to HDX-MS analysis across three labeling time points (0.25 min, 10 min, 60 min).

The HDX profiles of hSERT with and without SEC purification were nearly identical (Supplementary Figure. 2). Only exceptions were two peptides spanning residues 89-94 and 89-95 when measured at 10 min and 60 min (Supplementary Figure. 2). This could be explained by an observed increase in carry-over for the two peptides in the latest HDX-MS experiment. These locally-resolved control HDX-MS measurements are very sensitive to detect even modest local changes in SERT conformation between samples and we can

thus confidently conclude that the HDX-MS experiments we report are pertinent to a non-aggregated detergent:micellular solubilized state of hSERT.

Optimization of HDX-MS analysis for hSERT

During HDX-MS, a target protein is incubated with an excess of deuterated buffer for various time-intervals followed by quenching of the HDX reaction by lowering pH and temperature to approximately 2.5 and 0°C, respectively. The protein is subsequently digested using an acid-stable protease (e.g. pepsin) and resulting peptides are chromatographically separated and subjected to mass analysis by LC-MS. By monitoring the increase in mass over time (i.e., deuterium uptake) of peptic peptides covering the sequence of the target protein, the HDX of the protein can be measured in terms of local regions.

To be able to monitor the HDX of hSERT as comprehensively as possible, the sequence coverage (i.e. the coverage of the protein sequence by peptides generated by proteolysis) was optimized by evaluating the effect of quench buffer additives, the chain length of reverse phase chromatographic columns, use of different proteases, and the dwell time on the protease column.

The chaotropic agents guanidine hydrochloride (Gnd-HCl) and urea were tested and compared to quench buffer without any additives using immobilized pepsin for the digestion of the transporter. In agreement with our previous observations on another NSS family member[1], the addition of urea resulted in improvement of the sequence coverage. Intriguingly, the addition of Gnd-HCl led to a reduction in the sequence coverage, perhaps indicating that its charged nature is incompatible with this particular membrane transporter protein. The addition of detergent to the quench buffer (DDM, 1 mM) did not result in a large increase in the sequence coverage but it allowed for the identification of certain transmembrane regions not seen in the absence of detergent and was, therefore, included in the final quench conditions. The addition of TCEP to the quench buffer did not prove beneficial and the reducing agent, therefore, not included in the final quench buffer.

A comparison between digestions using pepsin, rhizopuspepsin, nepenthesin I, or nepenthesin II, was performed to identify the optimal aspartic protease for the HDX-MS analysis of hSERT. In a digestion screening, digestion by immobilized rhizopuspepsin resulted in the highest sequence coverage (58.3%) of hSERT. In contrast, digestion using immobilized pepsin resulted in 38.1% sequence coverage making the benefit of using the alternative protease apparent.

Upon selecting immobilized rhizopuspepsin for the HDX-MS analysis we identified that placing the protease column at 20°C and using a 150 µl/min flow rate (dwell time: approx. 4 seconds) was preferable. Attempts to cool the protease column to 0°C in hope of decreased back exchange or increasing the dwell time had negative effect on the sequence coverage. Decreasing the dwell time further by increasing the desalting flow rate, was not feasible as a 150 µl/min flow rate resulted in a back-pressure of 2000-2300 psi while the maximum recommended pressure for POROS 20AL resin is around 2500 psi.

The final peptide list consisted of 62 peptides, covering 69.1% of the protein sequence and was achieved by combining identified peptides from short and long chromatographic gradient methods where the peptides were identified by MS/MS acquired in either DIA or DDA manner (Supplementary Figure 2).

Supplementary Figures

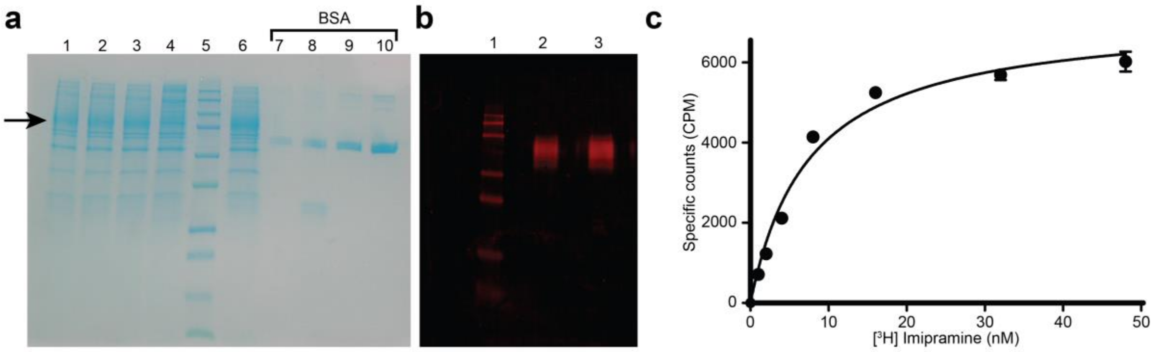

Supplementary Figure 1. Purified active hSERT. (**a**) SDS-PAGE gel with coomassie stain of purified samples of glycosylated hSERT (approx. 20-25% purity) separated by a single step nickel affinity purification was deemed sufficient for HDX-MS analysis. Lanes 1-4 and lane 6: Fractions containing purified hSERT. Lane 5: Kaleidoscope molecular weight ladder. Rows 7-10: BSA standard curve used for concentration determination of hSERT. The band representing hSERT (band broadness due to native glycan heterogeneity) is indicated by an arrow on the left side of the gel. (**b**) Western blot of Ni^{2+}-pure hSERT using hSERT antibody specific for the C-terminal of hSERT. Lane 1: Kaleidoscope molecular weight ladder. Lane 2: 1 µg of hSERT. Lane 3: 2 µg of hSERT. (**c**) Saturation binding experiment using [^{3}H]-imipramine was used to verify the activity of hSERT. The dissociation constant of the transporter to imipramine was determined as 7.5 +/- 0.7 nM. Unspecific binding was determined and corrected for using paroxetine. Standard deviations are shown as error bars but are in most cases too small to see. Source data can be found in Source data file.

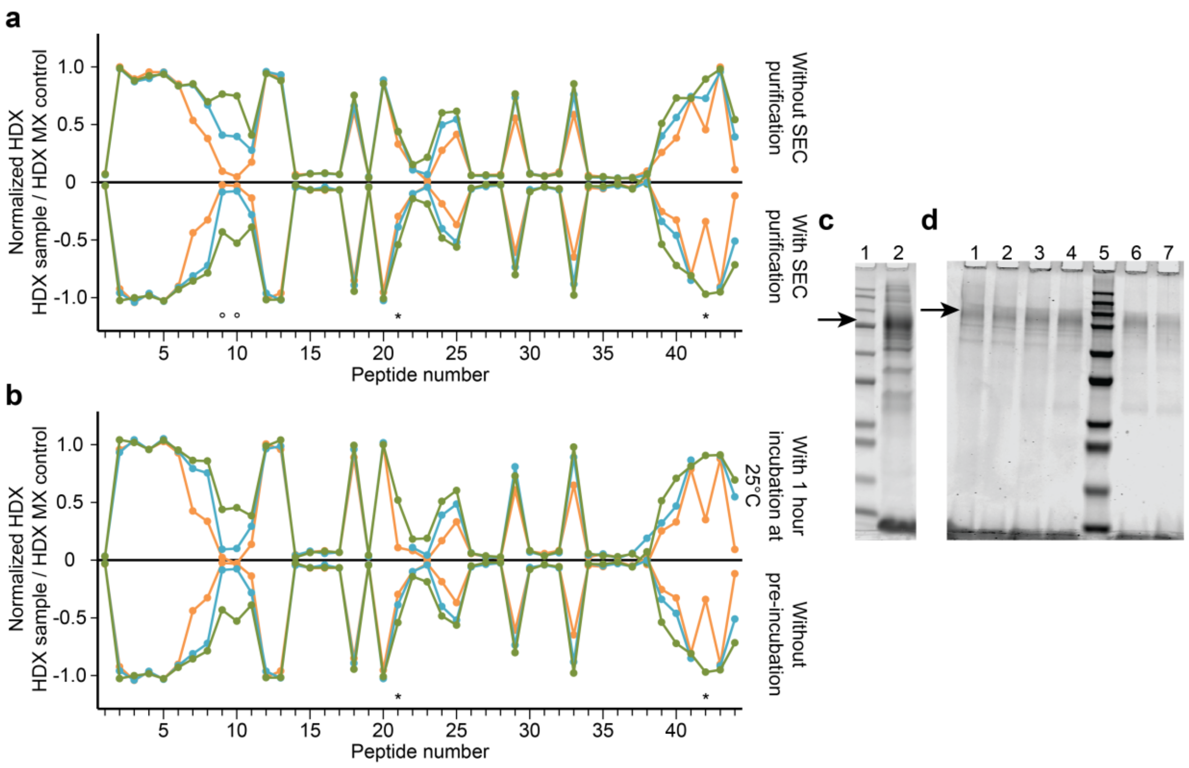

Supplementary Figure 2. Comparing the HDX of different hSERT samples. HDX of SERT following different sample treatments was measured over selected measuring time points (orange – 0.25 min; cyan – 10 min; green – 1 hour). (**a**) A comparison between the HDX of hSERT after Ni^{2+} affinity chromatography followed by dialysis (top, positive values), and hSERT after subsequent size-exclusion chromatography (SEC) (bottom, negative values). (**b**) HDX of hSERT after SEC (bottom, negative values) relative to similar sample only incubated at 25°C for 1 hour prior to HDX (top, positive values). (°) indicates differences in the fractional HDX between the two states, which could be due to carry over. (*) marks peptides missing from at least one of the states being compared. Normalized values are obtained through dividing the measured deuterium uptake of each peptide with the experimentally determined maximum deuterium uptake (MX control). Negative values are obtained by simple multiplication ("Normalized HDX value" * (-1)) to obtain the mirror image of a butterfly plot. (**c**) SDS-PAGE gel with coomassie stain of hSERT following Ni^{2+} affinity chromatography. Lane 1: Kaleidoscope molecular weight ladder. Lane 2: Pooled sample of hSERT from the most concentrated fractions collected following Ni^{2+} affinity chromatography (i.e. representative of the purity of hSERT samples used to acquire all the HDX-MS data described in the main text). (**d**) SDS-PAGE gel with coomassie stain of hSERT following Ni^{2+} affinity- and size exclusion chromatography. Lanes 1-4 and lanes 6-7: Fractions collected from a peak corresponding to the elution volume of monomeric hSERT. These fractions were pooled, up-concentrated, and used to acquire the HDX-MS data shown in the control experiment. Lane 5: Kaleidoscope molecular weight ladder. The band representing hSERT is indicated with an arrow on the left side of the gels in (**c**) and (**d**). Note, that the broadness of the band is expected due to the glycosylation of hSERT. Source data for the HDX data and uncropped SDS-PAGE, are provided as a Source data file.

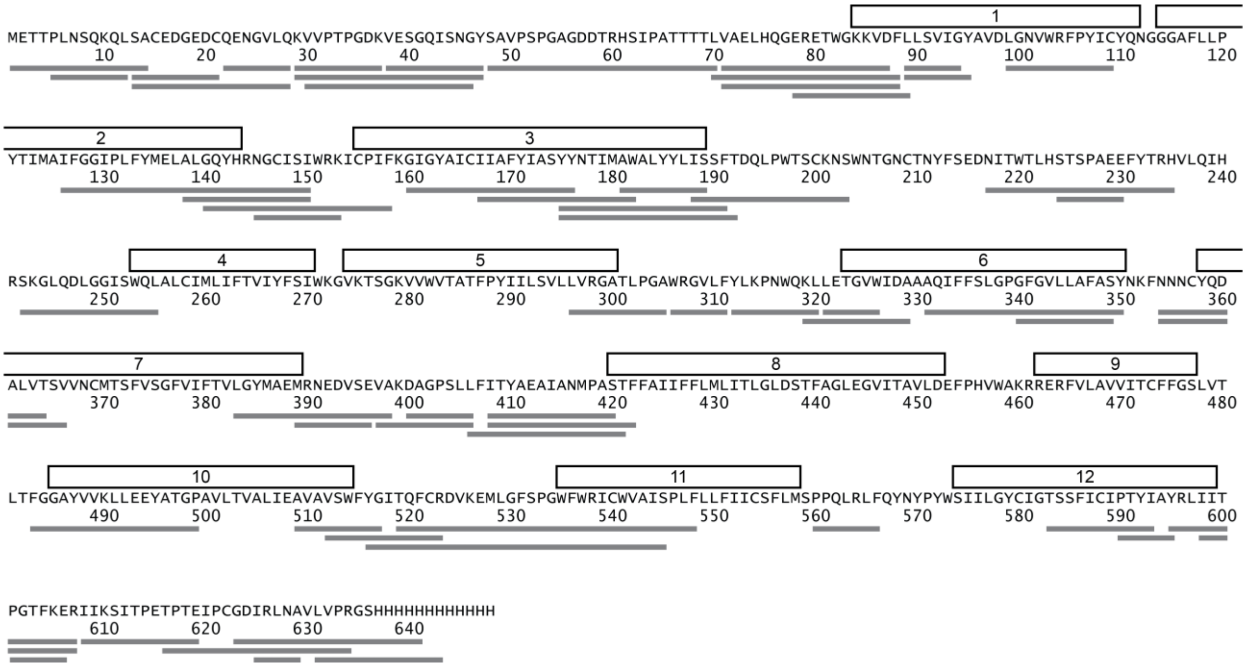

Supplementary Figure 3. Sequence coverage for HDX-MS analyses of hSERT. 62 peptides (illustrated as gray bars) covering 69.1% of the protein sequence, were identified following digestion of hSERT with immobilized rhizopuspepsin. Transmembrane helices are indicated above their respective sequence.

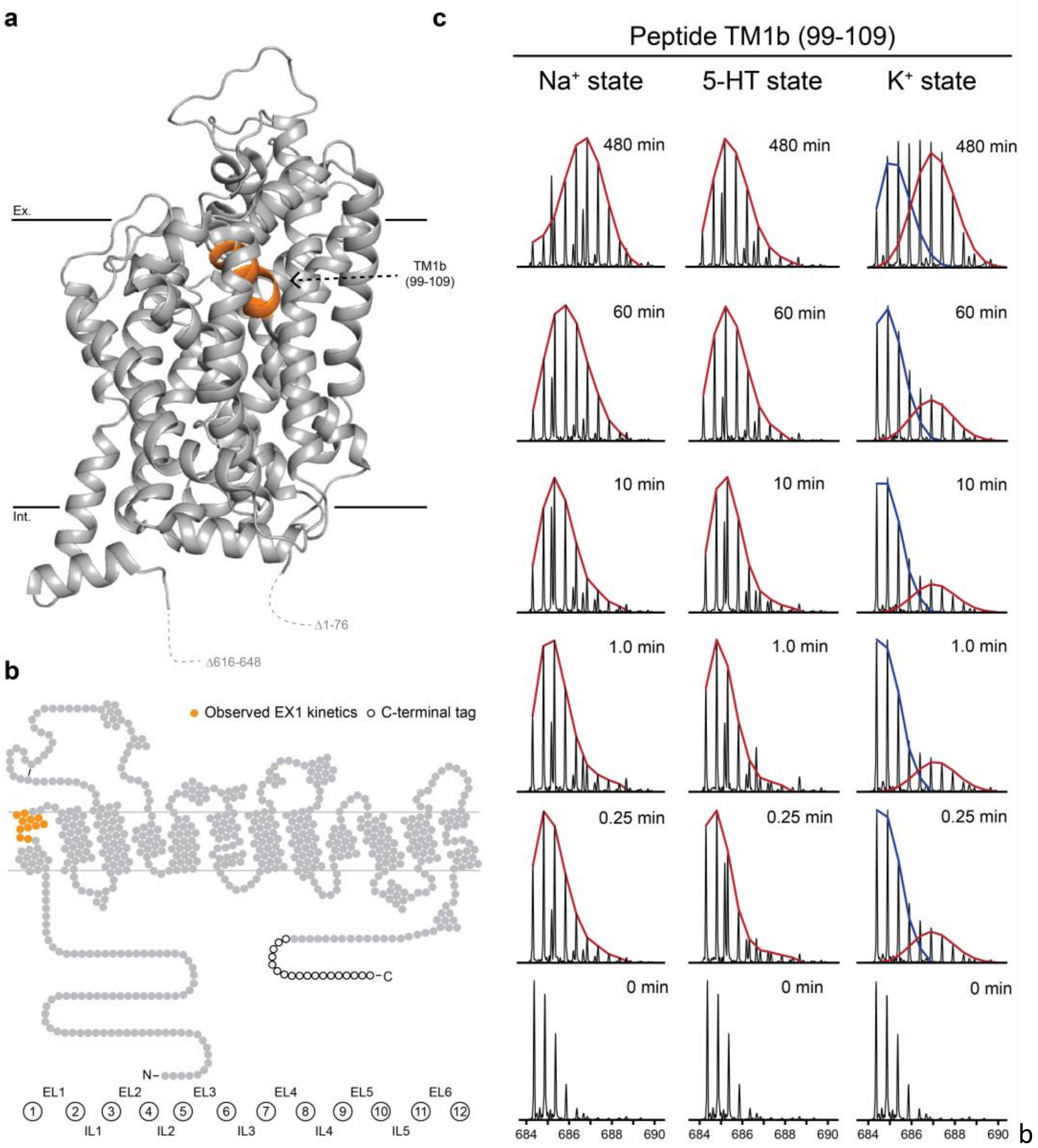

Supplementary Figure 4. Correlated exchange observed in TM1b. (**a**,**b**) The position of the segment covering TM1b (orange) illustrated on (**a**) a crystal structure (PDB: 5i75) and (**b**) on a snake diagram of hSERT. Grey dotted lines in the crystal structure indicate regions not included in the crystal structure. (**c**) Representative mass spectra for peptide 99-109 located in TM1b. In the presence of Na^+ and 5-HT, a single binominal isotope distribution (shown red) indicated that the residues in the segment underwent uncorrelated exchange (i.e. EX2 kinetics). In the presence of K^+, bimodal isotope distribution indicated that residues in the segment underwent correlated exchange. Spectra with visible low-mass (shown blue) and high mass (shown red) populations were fitted with HX-Express[2,3].

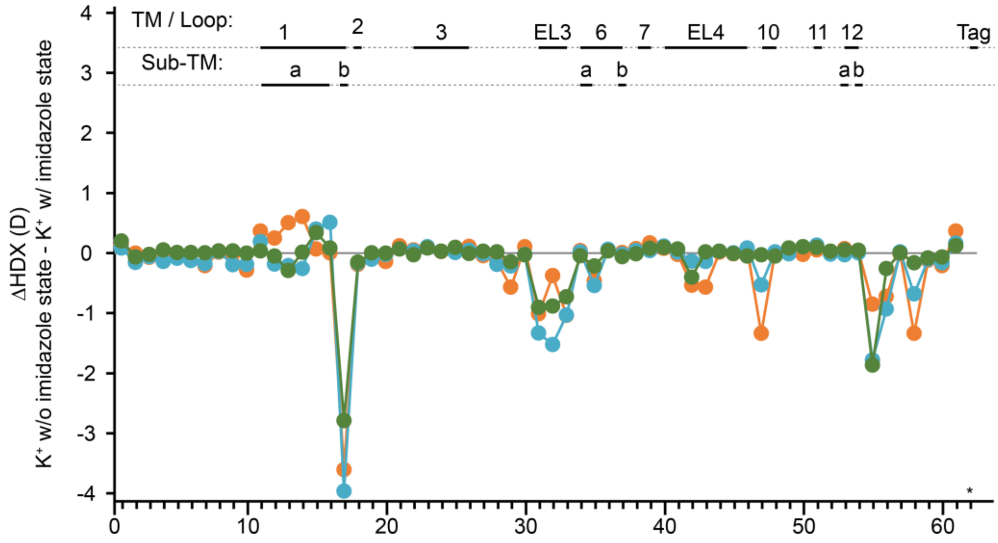

Supplementary Figure 5. Comparison of the HDX of hSERT in the presence of K^+ and imidazole with K^+. (**a**) A difference plot illustrating the difference in HDX when hSERT is in K^+ buffer in the presence or absence of imidazole (36 mM) for the 62 peptides identified from hSERT over the measured time points (orange – 0.25 min; cyan - 10 min; green - 1 hour; n = 1-2). Positive and negative values indicate decreased or increased HDX, respectively, when hSERT is in the presence of imidazole and K^+ compared to K^+ only. The peptides are arranged according to their position from the N- to C-terminal (Supplementary Table 1-3 for peptide order). (*) marks peptides where information was not available. Source data for the HDX data are provided as a Source data file.

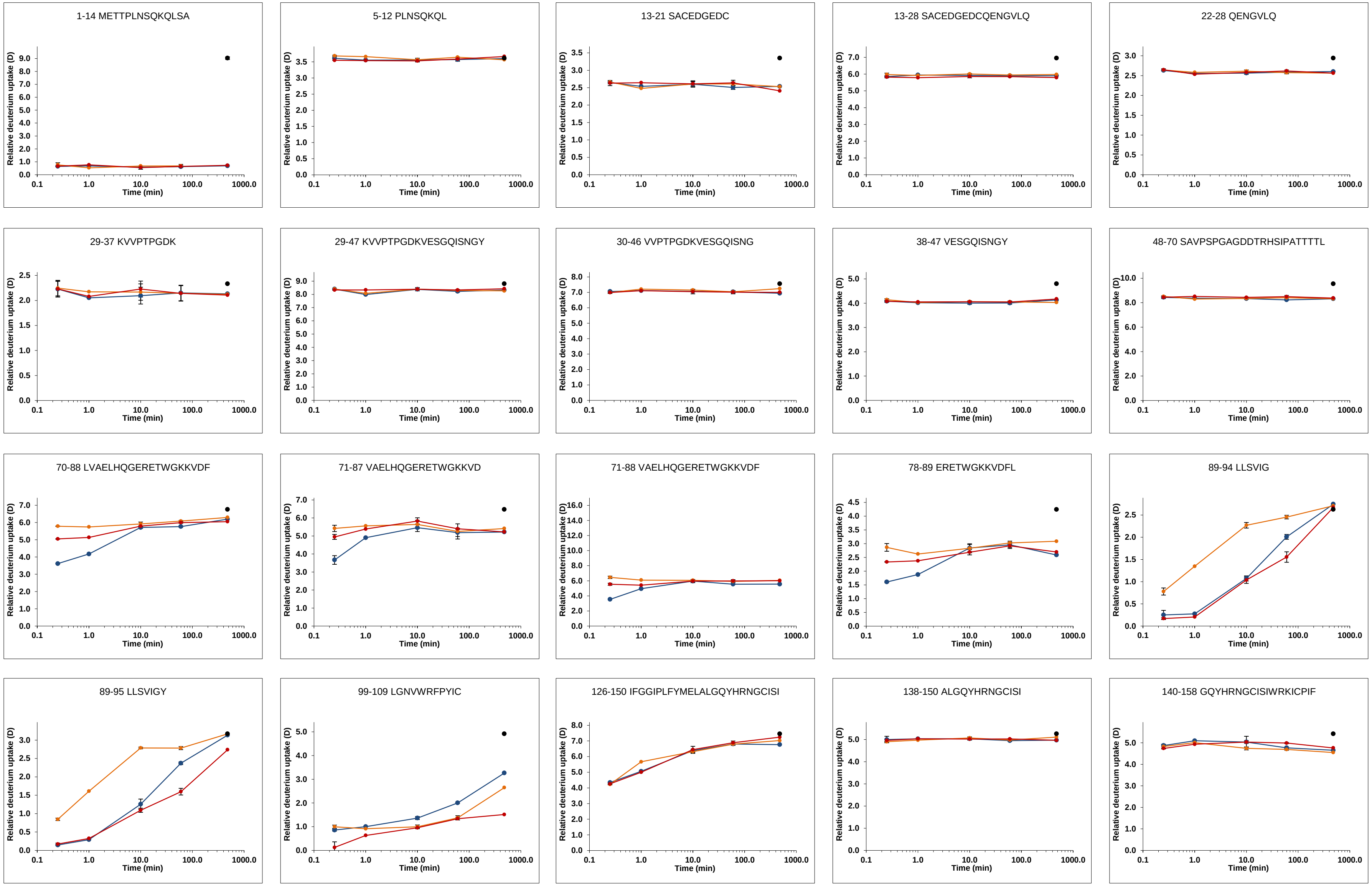
1-14 METTPLNSQKQLSA
5-12 PLNSQKQL
13-21 SACEDGEDC
13-28 SACEDGEDCQENGVLQ
22-28 QENGVLQ
29-37 KVVPTPGDK
29-47 KVVPTPGDKVESGQISNGY
30-46 VVPTPGDKVESGQISNG
38-47 VESGQISNGY
48-70 SAVPSPGAGDDTRHSIPATTTTL
70-88 LVAELHQGERETWGKKVDF
71-87 VAELHQGERETWGKKVD
71-88 VAELHQGERETWGKKVDF
78-89 ERETWGKKVDFL
89-94 LLSVIG
89-95 LLSVIGY
99-109 LGNVWRFPYIC
126-150 IFGGIPLFYMELALGQYHRNGCISI
138-150 ALGQYHRNGCISI
140-158 GQYHRNGCISIWRKICPIF
Relative deuterium uptake (D)
Time (min)

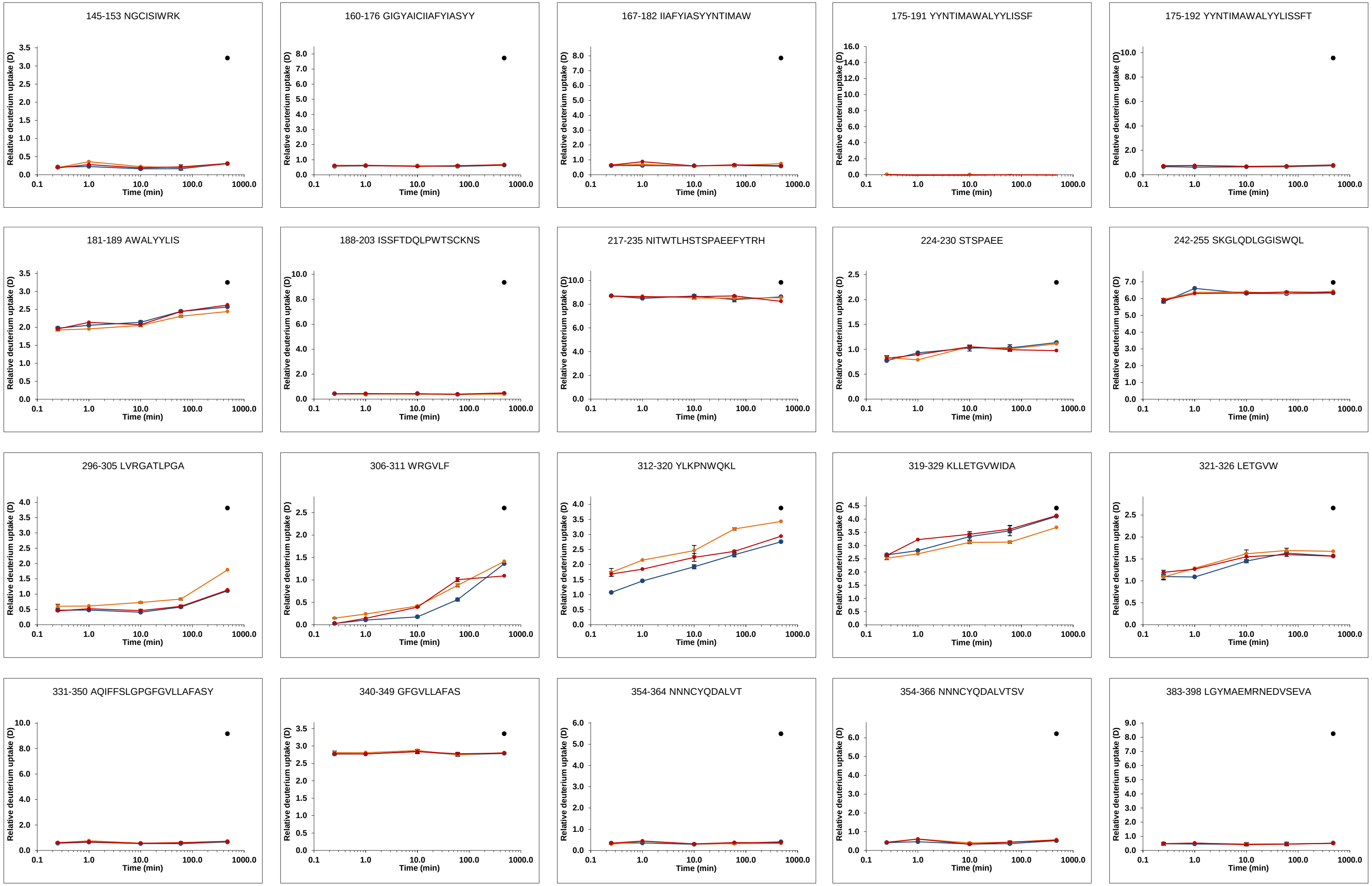
145-153 NGCISIWRK
160-176 GIGYAICIIAFYIASYY
167-182 IIAFYIASYYNTIMAW
175-191 YYNTIMAWALYYLISSF
175-192 YYNTIMAWALYYLISSFT
181-189 AWALYYLIS
188-203 ISSFTDQLPWTSCKNS
217-235 NITWTLHSTSPAEEFYTRH
224-230 STSPAEE
242-255 SKGLQDLGGISWQL
296-305 LVRGATLPGA
306-311 WRGVLF
312-320 YLKPNWQKL
319-329 KLLETGVWIDA
321-326 LETGVW
331-350 AQIFFSLGPGFGVLLAFASY
340-349 GFGVLLAFAS
354-364 NNNCYQDALVT
354-366 NNNCYQDALVTSV
383-398 LGYMAEMRNEDVSEVA
Relative deuterium uptake (D)
Time (min)

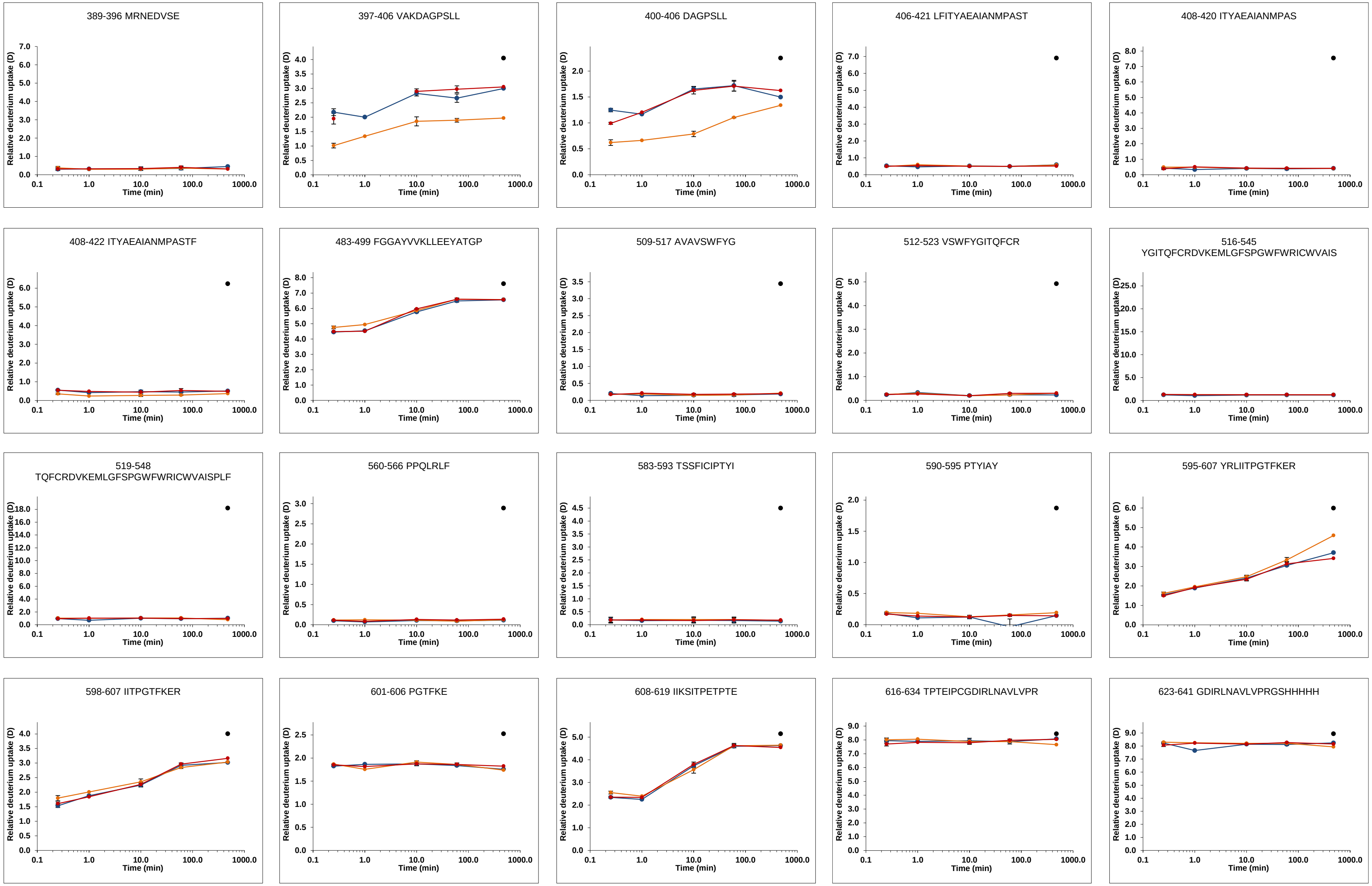
389-396 MRNEDVSE
397-406 VAKDAGPSLL
400-406 DAGPSLL
406-421 LFITYAEAIANMPAST
408-420 ITYAEAIANMPAS
408-422 ITYAEAIANMPASTF
483-499 FGGAYVVKLLEEYATGP
509-517 AVAVSWFYG
512-523 VSWFYGITQFCR
516-545 YGITQFCRDVKEMLGFSPGWFWRICWVAIS
519-548 TQFCRDVKEMLGFSPGWFWRICWVAISPLF
560-566 PPQLRLF
583-593 TSSFICIPTYI
590-595 PTYIAY
595-607 YRLIITPGTFKER
598-607 IITPGTFKER
601-606 PGTFKE
608-619 IIKSITPETPTE
616-634 TPTEIPCGDIRLNAVLVPR
623-641 GDIRLNAVLVPRGSHHHHH
Relative deuterium uptake (D)
Time (min)

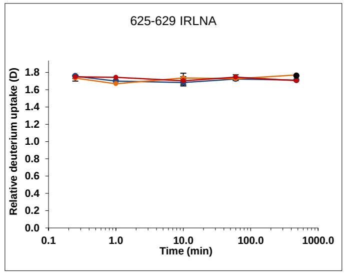

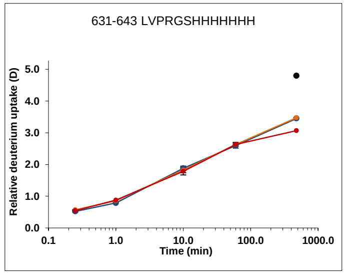

Supplementary Figure 6. Deuterium uptake plots for HDX experiment of hSERT in the presence or absence of Na^+, K^+, and 5-HT. The relative deuterium uptake is plotted as a function of labeling time (0.25 min, 1 min, 10 min, 60 min, 480 min) for the 62 peptides used in this experiment. Blue, orange, and red, plotted lines represent the HDX of hSERT in Na^+, K^+, and 5-HT states, respectively. Black dot at 480 min, shows the HDX of maximum labeled sample (n = 1). Standard deviations are plotted as error bars for the 0.25 minutes, 10 minutes and 60 minutes labeling points (n = 2 - 4). Maximum labeled controls are absent for peptides 13 (residues 71-88), 24 (residues 175-191), 41 (residues 389-396), and 50 (residues 516-545). Source data for the HDX data are provided as a Source data file.

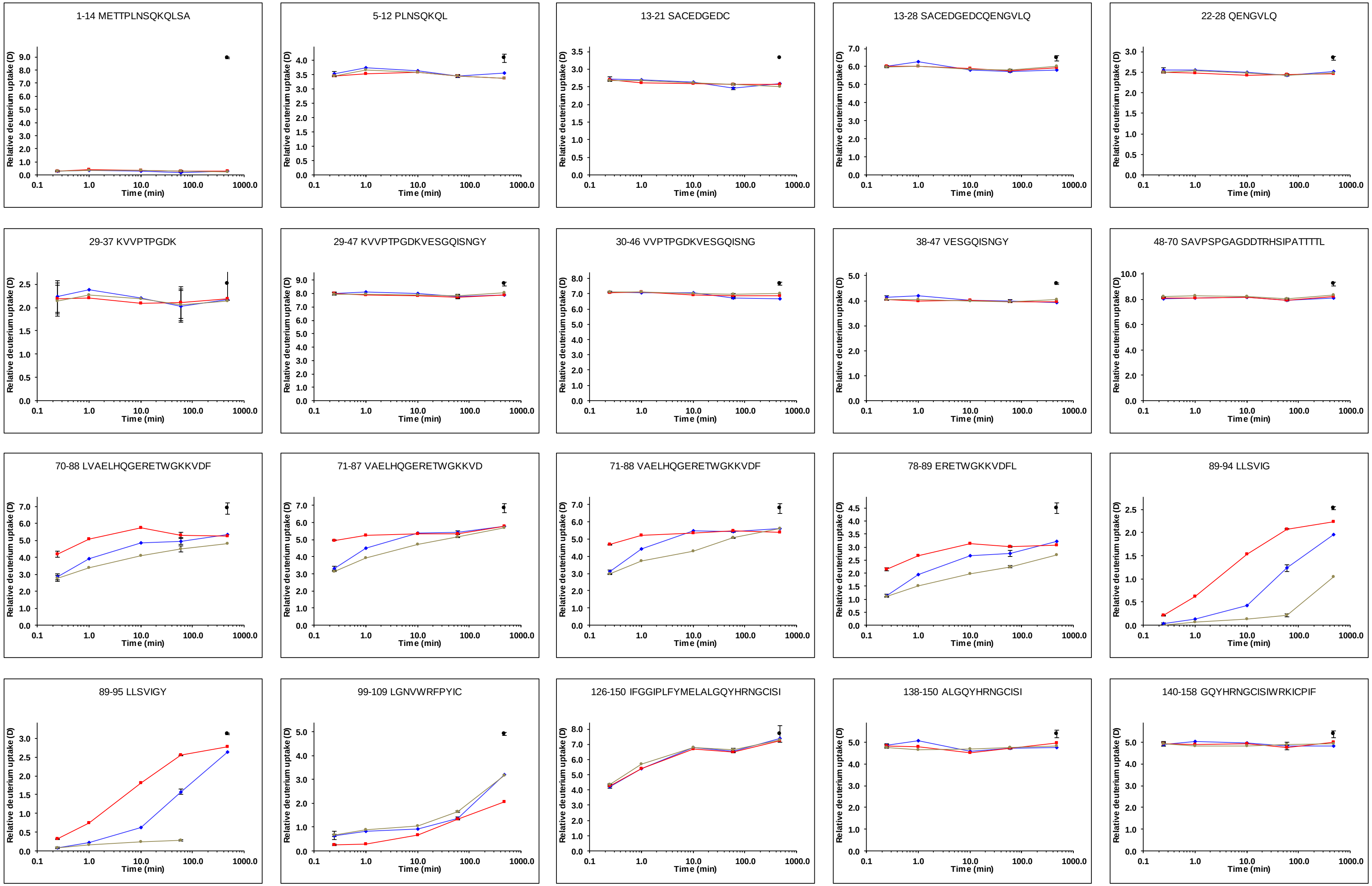
1-14 METTPLNSQKQLSA
5-12 PLNSQKQL
13-21 SACEDGEDC
13-28 SACEDGEDCQENGVLQ
22-28 QENGVLQ
29-37 KVVPTPGDK
29-47 KVVPTPGDKVESGQISNGY
30-46 VVPTPGDKVESGQISNG
38-47 VESGQISNGY
48-70 SAVPSPGAGDDTRHSIPATTTTL
70-88 LVAELHQGERETWGKKVDF
71-87 VAELHQGERETWGKKVD
71-88 VAELHQGERETWGKKVDF
78-89 ERETWGKKVDFL
89-94 LLSVIG
89-95 LLSVIGY
99-109 LGNVWRFPYIC
126-150 IFGGIPLFYMELALGQYHRNGCISI
138-150 ALGQYHRNGCISI
140-158 GQYHRNGCISIWRKICPIF
Relative deuterium uptake (D)
Time (min)

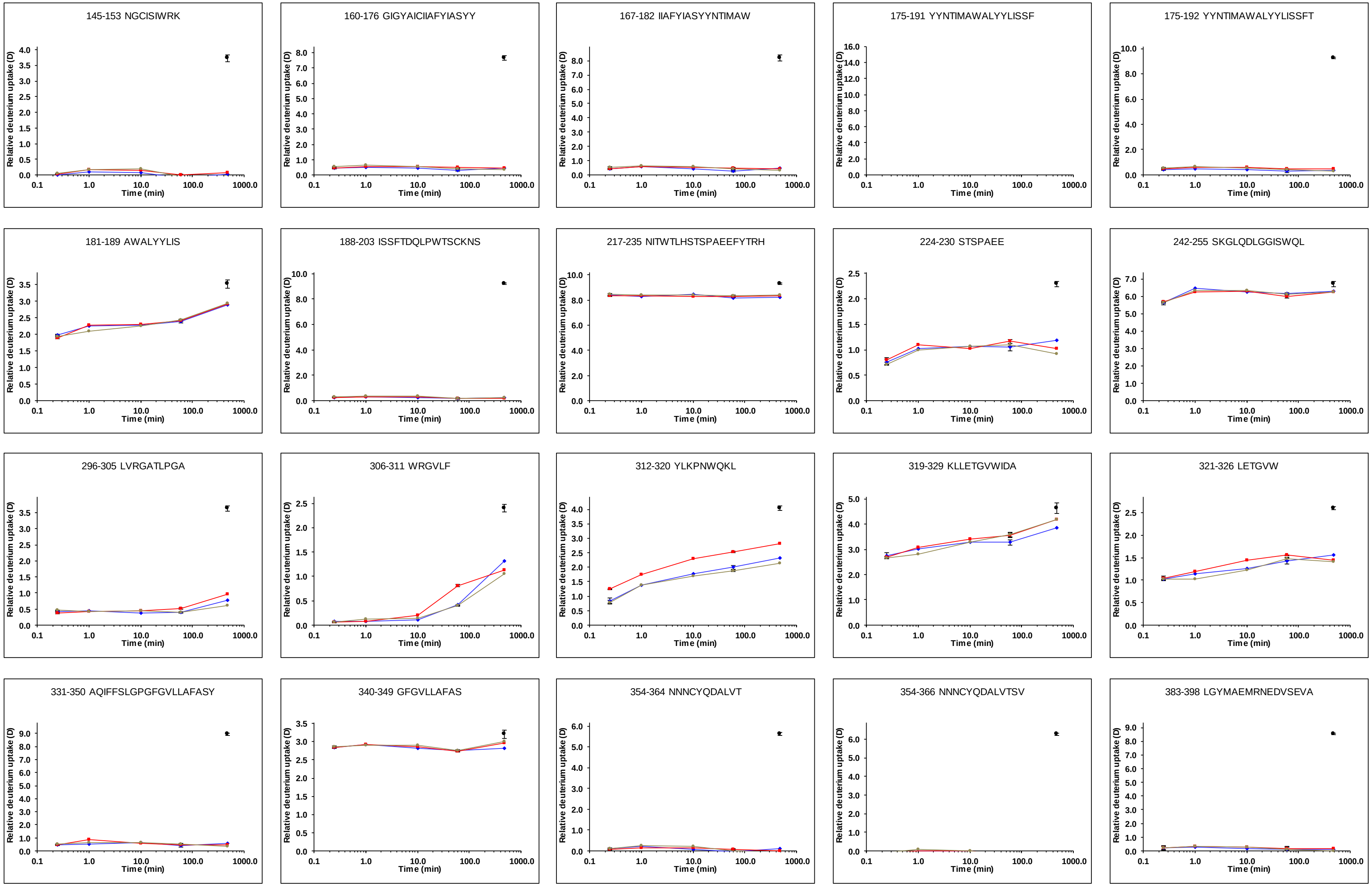
145-153 NGCISIWRK
160-176 GIGYAICIIAFYIASYY
167-182 IIAFYIASYYNTIMAW
175-191 YYNTIMAWALYYLISSF
175-192 YYNTIMAWALYYLISSFT
181-189 AWALYYLIS
188-203 ISSFTDQLPWTSCKNS
217-235 NITWTLHSTSPAEEFYTRH
224-230 STSPAEE
242-255 SKGLQDLGGISWQL
296-305 LVRGATLPGA
306-311 WRGVLF
312-320 YLKPNWQKL
319-329 KLLETGVWIDA
321-326 LETGVW
331-350 AQIFFSLGPGFGVLLAFASY
340-349 GFGVLLAFAS
354-364 NNNCYQDALVT
354-366 NNNCYQDALVTSV
383-398 LGYMAEMRNEDVSEVA
Relative deuterium uptake (D)
Time (min)

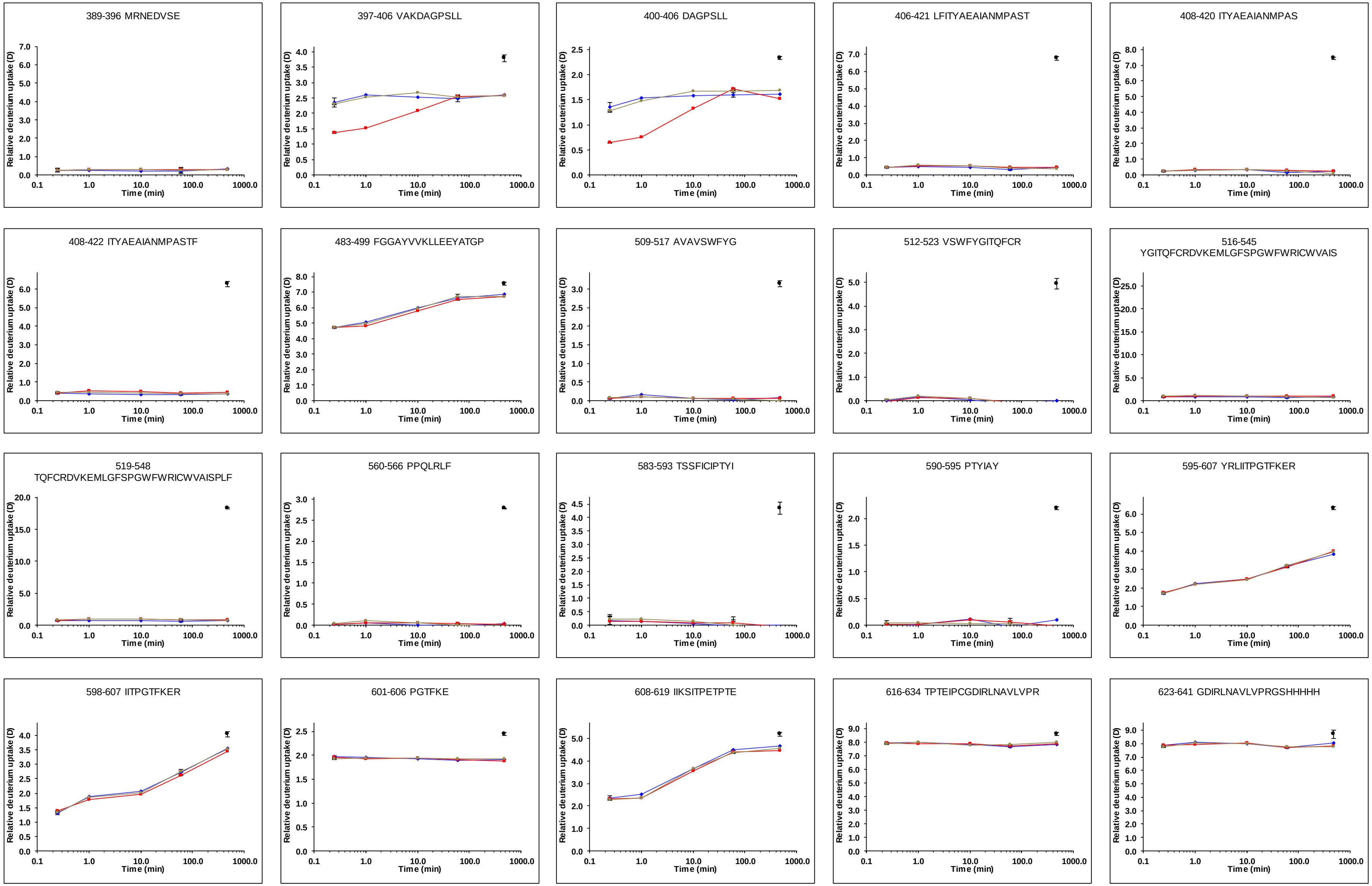
389-396 MRNEDVSE
397-406 VAKDAGPSLL
400-406 DAGPSLL
406-421 LFITYAEAIANMPAST
408-420 ITYAEAIANMPAS
408-422 ITYAEAIANMPASTF
483-499 FGGAYVVKLLEEYATGP
509-517 AVAVSWFYG
512-523 VSWFYGITQFCR
516-545
YGITQFCRDVKEMLGFSPGWFWRICWVAIS
519-548
TQFCRDVKEMLGFSPGWFWRICWVAISPLF
560-566 PPQLRLF
583-593 TSSFICIPTYI
590-595 PTYIAY
595-607 YRLIITPGTFKER
598-607 IITPGTFKER
601-606 PGTFKE
608-619 IIKSITPETPTE
616-634 TPTEIPCGDIRLNAVLVPR
623-641 GDIRLNAVLVPRGSHHHHH
Relative deuterium uptake (D)
Time (min)

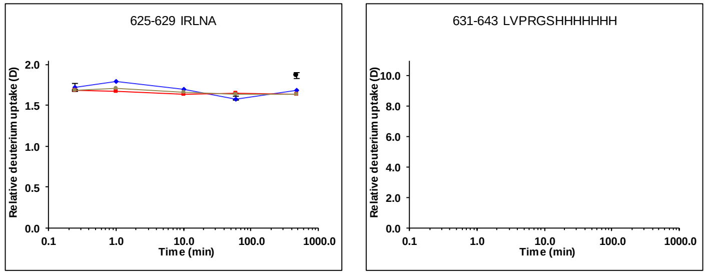

Supplementary Figure 7. Deuterium uptake plots for HDX experiment of hSERT in the presence or absence of Na^+, cocaine and ibogaine. The relative deuterium uptake is plotted as a function of labeling time (0.25 min, 1 min, 10 min, 60 min, 480 min) for the 62 peptides used in this experiment. Blue, red, and grey, plotted lines represent the HDX of hSERT in Na^+, ibogaine, and cocaine states, respectively. Black dot at 480 min shows the HDX of maximum labeled sample (n = 6). Standard deviations are plotted as error bars for the 0.25 minutes and 60 minutes labeling points (n = 2). The HDX of peptides 24 (residues 175-191) and 62 (residues 631-643) were not successfully collected for these states. Maximum labeled controls are absent for peptides 41 (residues 389-396) and 50 (residues 516-545), and error bars are absent for the maximum labeled control for peptide 3 (residues 13-21). Source data for the HDX data are provided as a Source data file.

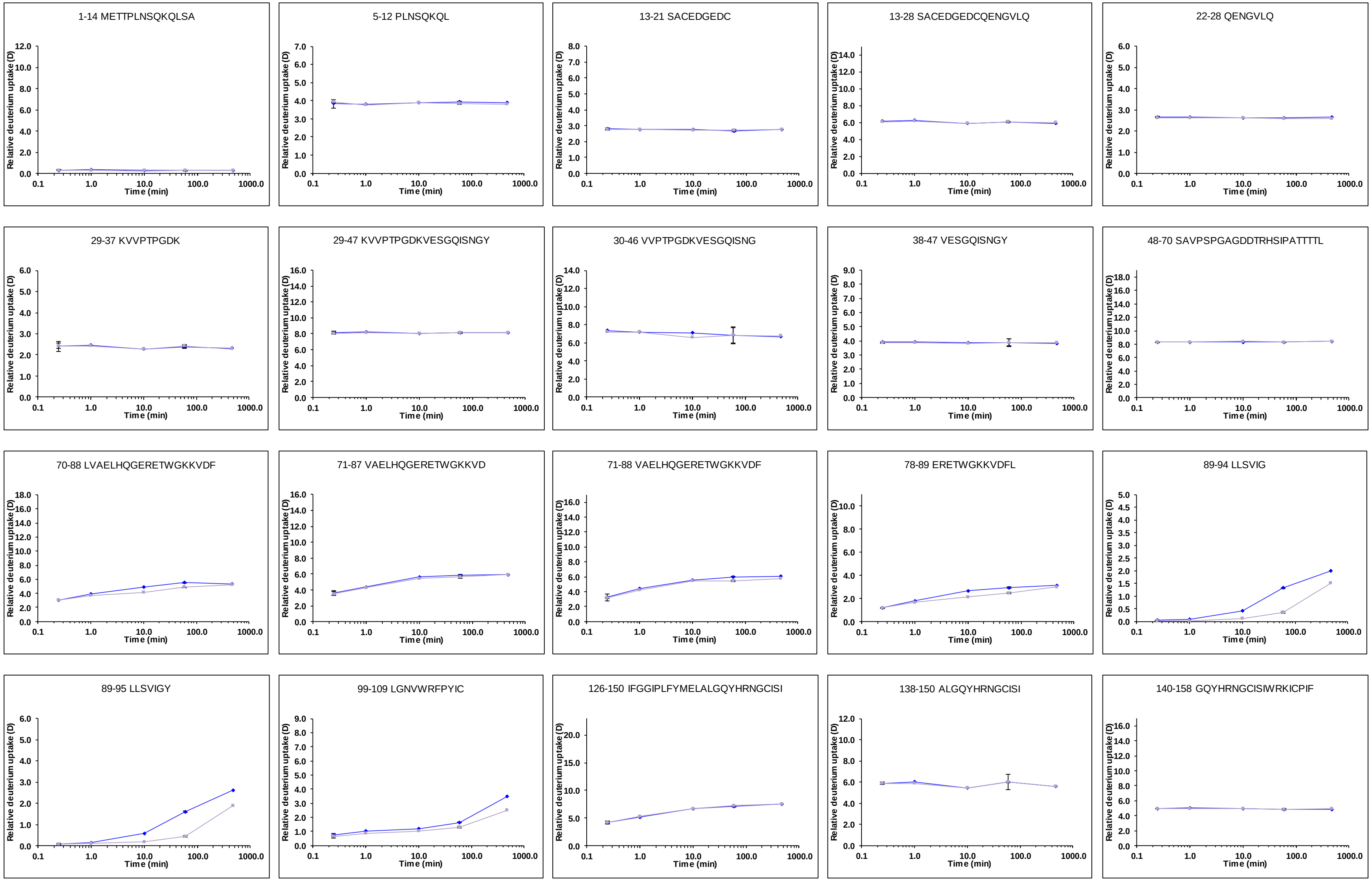
1-14 METTPLNSQKQLSA
5-12 PLNSQKQL
13-21 SACEDGEDC
13-28 SACEDGEDCQENGVLQ
22-28 QENGVLQ
29-37 KVVPTPGDK
29-47 KVVPTPGDKVESGQISNGY
30-46 VVPTPGDKVESGQISNG
38-47 VESGQISNGY
48-70 SAVPSPGAGDDTRHSIPATTTTL
70-88 LVAELHQGERETWGKKVDF
71-87 VAELHQGERETWGKKVD
71-88 VAELHQGERETWGKKVDF
78-89 ERETWGKKVDFL
89-94 LLSVIG
89-95 LLSVIGY
99-109 LGNVWRFPYIC
126-150 IFGGIPLFYMELALGQYHRNGCISI
138-150 ALGQYHRNGCISI
140-158 GQYHRNGCISIWRKICPIF
Relative deuterium uptake (D)
Time (min)

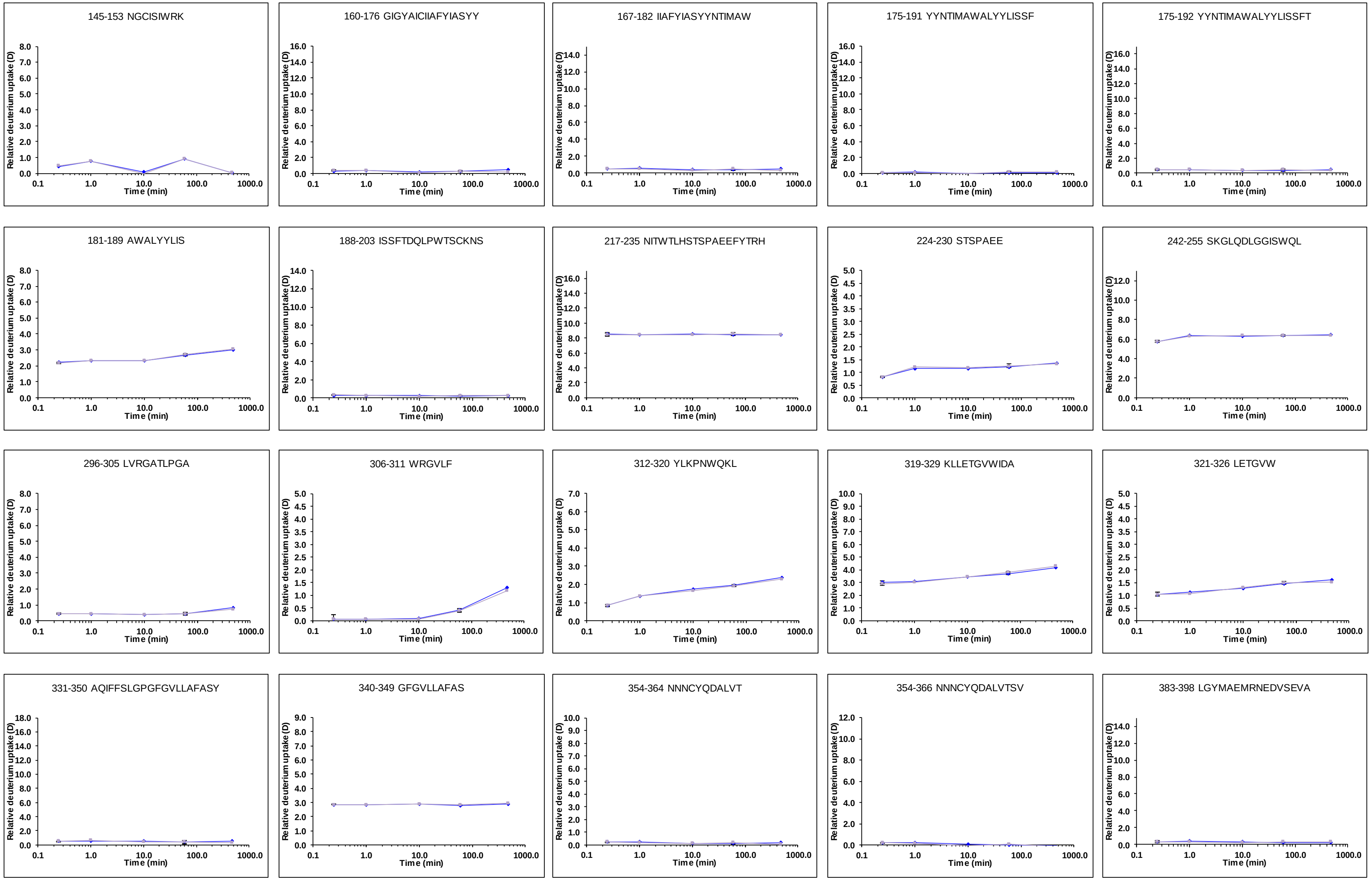
145-153 NGCISIWRK
160-176 GIGYAICIIAFYIASYY
167-182 IIAFYIASYYNTIMAW
175-191 YYNTIMAWALYYLISSF
175-192 YYNTIMAWALYYLISSFT
181-189 AWALYYLIS
188-203 ISSFTDQLPWTSCKNS
217-235 NITWTLHSTSPAEEFYTRH
224-230 STSPAEE
242-255 SKGLQDLGGISWQL
296-305 LVRGATLPGA
306-311 WRGVLF
312-320 YLKPNWQKL
319-329 KLLETGVWIDA
321-326 LETGVW
331-350 AQIFFSLGPGFGVLLAFASY
340-349 GFGVLLAFAS
354-364 NNNCYQDALVT
354-366 NNNCYQDALVTSV
383-398 LGYMAEMRNEDVSEVA
Relative deuterium uptake (D)
Time (min)

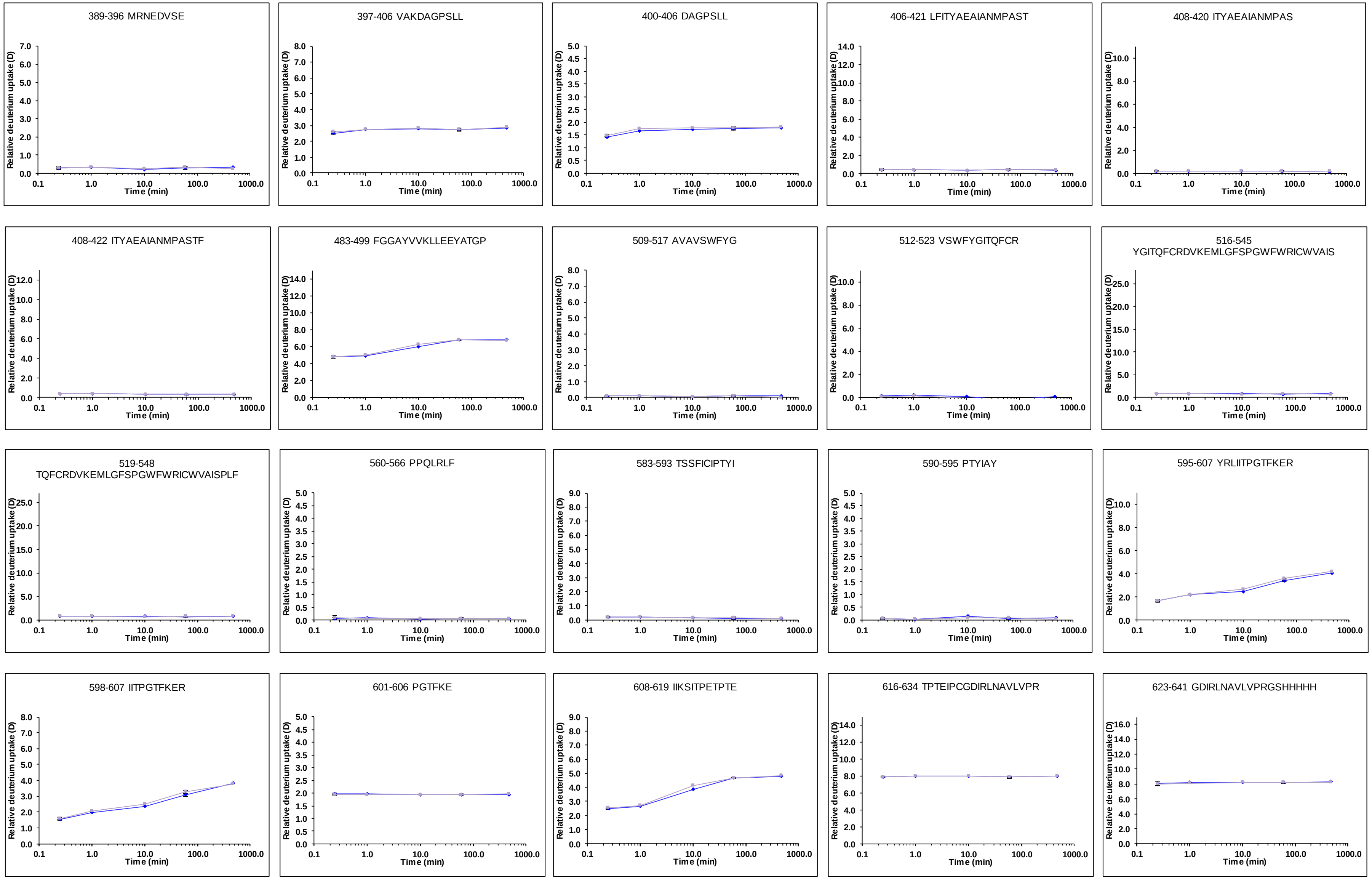
389-396 MRNEDVSE
397-406 VAKDAGPSLL
400-406 DAGPSLL
406-421 LFITYAEAIANMPAST
408-420 ITYAEAIANMPAS
408-422 ITYAEAIANMPASTF
483-499 FGGAYVVKLLEEYATGP
509-517 AVAVSWFYG
512-523 VSWFYGITQFCR
516-545 YGITQFCRDVKEMLGFSPGWFWRICWVAIS
519-548 TQFCRDVKEMLGFSPGWFWRICWVAISPLF
560-566 PPQLRLF
583-593 TSSFICIPTYI
590-595 PTYIAY
595-607 YRLIITPGTFKER
598-607 IITPGTFKER
601-606 PGTFKE
608-619 IIKSITPETPTE
616-634 TPTEIPCGDIRLNAVLVPR
623-641 GDIRLNAVLVPRGSHHHHH
Relative deuterium uptake (D)
Time (min)

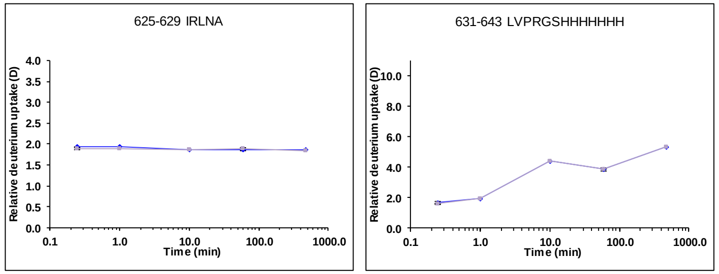

Supplementary Figure 8. Deuterium uptake plots for HDX experiment of hSERT in the presence or absence of Na^+ and S-citalopram. The relative deuterium uptake is plotted as a function of labeling time (0.25 min, 1 min, 10 min, 60 min, 480 min) for the 62 peptides used in this experiment. Blue and purple plotted lines represent the HDX of hSERT in Na^+ and S-citalopram states, respectively. Standard deviations are plotted as error bars for the 0.25 minutes and 60 minutes labeling points (n = 3). Maximum labeled controls are absent for all peptides comparing Na^+ and S-citalopram. Source data for the HDX data are provided as a Source data file.

Supplementary tables

Full-scale excel versions of all Supplementary Tables can be found in Supplementary Dataset 6.

Supplementary Table 1. The relative deuterium uptake measured for Na+, K+, and 5-HT states at all labelling time points (0.25 min – 480 min) for the 62 peptides identified from hSERT along with the theoretical maximum uptake and the measured maximum uptake

Experiment: Na^+, K^+, 5-HT					Relative deuterium uptake														
					Na^+ state					K^+ state					5-HT state				
Peptide number	Residues	Sequence	Theoretical uptake	Maximum uptake	0.25	1 min	10 min	60 min	480 min	0.25 min	1 min	10 min	60 min	480 min	0.25 min	1 min	10 min	60 min	480 min
1	1-14	METTPLNSQKQLSA	12 D	9.03 D	0.65 +/- 0.004 D	0.68 D	0.58 +/- 0.038 D	0.62 +/- 0.050 D	0.69 D	0.77 +/- 0.158 D	0.54 D	0.67 +/- (N/A)	0.69 +/- 0.117 D	(N/A)	0.66 +/- 0.037 D	0.76 D	0.55 +/- 0.127 D	0.64 +/- 0.079 D	0.72 D
2	5-12	PLNSQKQL	7 D	3.61 D	3.61 +/- 0.009 D	3.55 D	3.55 +/- 0.058 D	3.57 +/- 0.053 D	3.60 D	3.68 +/- 0.022 D	3.66 D	3.56 +/- 0.009 D	3.64 +/- 0.005 D	3.56 D	3.55 +/- 0.000 D	3.54 D	3.53 +/- 0.024 D	3.59 +/- 0.056 D	3.67 D
3	13-21	SACEDGEDC	8 D	3.35 D	2.65 +/- 0.038 D	2.54 D	2.60 +/- 0.084 D	2.51 +/- 0.054 D	2.54 D	2.66 +/- 0.010 D	2.48 D	2.61 +/- 0.089 D	2.61 +/- 0.055 D	2.53 D	2.63 +/- 0.074 D	2.64 D	2.61 +/- 0.065 D	2.64 +/- 0.078 D	2.41 D
4	13-28	SACEDGEDCQENGVLQ	15 D	6.95 D	5.85 +/- 0.019 D	5.95 D	5.93 +/- 0.050 D	5.90 +/- 0.027 D	5.90 D	5.98 +/- 0.081 D	5.92 D	6.01 +/- 0.031 D	5.94 +/- 0.032 D	5.98 D	5.82 +/- 0.042 D	5.78 D	5.86 +/- 0.079 D	5.85 +/- 0.024 D	5.79 D
5	22-28	QENGVLQ	6 D	2.94 D	2.63 +/- 0.004 D	2.55 D	2.56 +/- 0.032 D	2.59 +/- 0.021 D	2.60 D	2.64 +/- 0.029 D	2.58 D	2.61 +/- 0.031 D	2.57 +/- 0.030 D	2.56 D	2.65 +/- 0.001 D	2.54 D	2.58 +/- 0.013 D	2.62 +/- 0.024 D	2.56 D
6	29-37	KVVPTPGDK	6 D	2.33 D	2.23 +/- 0.155 D	2.05 D	2.10 +/- 0.168 D	2.15 +/- 0.155 D	2.13 D	2.25 +/- 0.155 D	2.17 D	2.17 +/- 0.164 D	2.14 +/- 0.155 D	2.12 D	2.23 +/- 0.162 D	2.08 D	2.23 +/- 0.159 D	2.14 +/- 0.155 D	2.11 D
7	29-47	KVVPTPGDKVESGQISNGY	16 D	8.80 D	8.40 +/- 0.024 D	7.99 D	8.38 +/- 0.014 D	8.23 +/- 0.070 D	8.32 D	8.40 +/- 0.016 D	8.07 D	8.39 +/- 0.123 D	8.30 +/- 0.068 D	8.26 D	8.33 +/- 0.015 D	8.33 D	8.39 +/- 0.007 D	8.33 +/- 0.022 D	8.42 D
8	30-46	VVPTPGDKVESGQISNG	14 D	7.55 D	7.05 +/- 0.055 D	7.11 D	7.06 +/- 0.018 D	7.03 +/- 0.076 D	6.94 D	6.99 +/- 0.060 D	7.21 D	7.15 +/- 0.051 D	7.03 +/- 0.061 D	7.24 D	6.99 +/- 0.028 D	7.10 D	7.04 +/- 0.140 D	7.01 +/- 0.108 D	7.00 D
9	38-47	VESGQISNGY	9 D	4.80 D	4.08 +/- 0.043 D	4.02 D	4.00 +/- 0.051 D	4.01 +/- 0.054 D	4.13 D	4.14 +/- 0.040 D	4.03 D	4.06 +/- 0.024 D	4.05 +/- 0.029 D	4.03 D	4.08 +/- 0.033 D	4.05 D	4.06 +/- 0.042 D	4.05 +/- 0.044 D	4.17 D
10	48-70	SAVPSPGAGDDTRHSIPATTTTL	19 D	9.54 D	8.44 +/- 0.045 D	8.37 D	8.34 +/- 0.043 D	8.23 +/- 0.027 D	8.32 D	8.52 +/- 0.047 D	8.30 D	8.35 +/- 0.015 D	8.41 +/- 0.023 D	8.32 D	8.45 +/- 0.103 D	8.51 D	8.43 +/- 0.033 D	8.49 +/- 0.103 D	8.38 D
11	70-88	LVAELHQGERETWGKKVDF	18 D	6.75 D	3.61 +/- 0.020 D	4.17 D	5.71 +/- 0.053 D	5.76 +/- 0.044 D	6.19 D	5.78 +/- 0.014 D	5.74 D	5.92 +/- 0.112 D	6.08 +/- 0.033 D	6.29 D	5.05 +/- 0.018 D	5.13 D	5.79 +/- 0.020 D	5.99 +/- 0.044 D	6.05 D
12	71-87	VAELHQGERETWGKKVD	16 D	6.47 D	3.67 +/- 0.242 D	4.90 D	5.46 +/- 0.212 D	5.19 +/- 0.217 D	5.22 D	5.42 +/- 0.173 D	5.56 D	5.64 +/- 0.230 D	5.25 +/- 0.423 D	5.41 D	4.95 +/- 0.135 D	5.39 D	5.83 +/- 0.180 D	5.40 +/- 0.037 D	5.23 D
13	71-88	VAELHQGERETWGKKVDF	17 D	D	3.54 +/- 0.014 D	4.95 D	5.96 +/- 0.205 D	5.55 +/- 0.038 D	5.56 D	6.45 +/- 0.157 D	6.09 D	6.06 +/- 0.027 D	5.92 +/- 0.122 D	6.05 D	5.54 +/- 0.128 D	5.41 D	5.96 +/- 0.173 D	5.98 +/- 0.165 D	6.00 D
14	78-89	ERETWGKKVDFL	11 D	4.24 D	1.61 +/- 0.026 D	1.88 D	2.84 +/- 0.125 D	2.96 +/- 0.095 D	2.59 D	2.86 +/- 0.140 D	2.62 D	2.83 +/- 0.163 D	3.02 +/- 0.067 D	3.09 D	2.34 +/- 0.007 D	2.38 D	2.69 +/- 0.096 D	2.92 +/- 0.095 D	2.70 D
15	89-94	LLSVIG	5 D	2.63 D	0.25 +/- 0.103 D	0.27 D	1.07 +/- 0.057 D	2.01 +/- 0.055 D	2.75 D	0.78 +/- 0.081 D	1.35 D	2.27 +/- 0.063 D	2.45 +/- 0.042 D	2.71 D	0.17 +/- 0.018 D	0.20 D	1.04 +/- 0.074 D	1.56 +/- 0.118 D	2.67 D
16	89-95	LLSVIGY	6 D	3.17 D	0.15 +/- 0.020 D	0.29 D	1.26 +/- 0.141 D	2.37 +/- 0.038 D	3.13 D	0.85 +/- 0.033 D	1.61 D	2.79 +/- 0.024 D	2.78 +/- 0.043 D	3.17 D	0.17 +/- 0.027 D	0.33 D	1.09 +/- 0.056 D	1.60 +/- 0.090 D	2.74 D
17	99-109	LGNVWRFPYIC	9 D	4.91 D	0.86 +/- 0.047 D	1.00 D	1.36 +/- 0.064 D	2.00 +/- 0.029 D	3.26 D	1.00 +/- 0.068 D	0.91 D	0.99 +/- 0.080 D	1.37 +/- 0.089 D	2.65 D	0.13 +/- 0.236 D	0.63 D	0.95 +/- 0.016 D	1.34 +/- 0.051 D	1.51 D
18	126-150	IFGGIPLFYMELALGQYHRNGCISI	23 D	7.45 D	4.34 +/- 0.034 D	5.06 D	6.38 +/- 0.089 D	6.80 +/- 0.084 D	6.76 D	4.23 +/- 0.030 D	5.67 D	6.32 +/- 0.109 D	6.80 +/- 0.068 D	7.03 D	4.26 +/- 0.057 D	5.00 D	6.45 +/- 0.211 D	6.88 +/- 0.113 D	7.24 D
19	138-150	ALGQYHRNGCISI	12 D	5.26 D	5.00 +/- 0.145 D	5.03 D	5.03 +/- 0.054 D	4.95 +/- 0.039 D	4.97 D	4.90 +/- 0.013 D	4.97 D	5.07 +/- 0.047 D	4.98 +/- 0.028 D	5.11 D	4.96 +/- 0.055 D	5.04 D	5.02 +/- 0.040 D	5.03 +/- 0.017 D	4.97 D
20	140-158	GQYHRNGCISIWRKICPIF	17 D	5.42 D	4.88 +/- 0.052 D	5.10 D	5.04 +/- 0.066 D	4.77 +/- 0.042 D	4.65 D	4.83 +/- 0.108 D	5.01 D	4.74 +/- 0.069 D	4.69 +/- 0.037 D	4.55 D	4.74 +/- 0.021 D	4.93 D	5.03 +/- 0.269 D	4.99 +/- 0.024 D	4.76 D
21	145-153	NGCISIWRK	8 D	3.21 D	0.21 +/- 0.002 D	0.23 D	0.16 +/- 0.025 D	0.17 +/- 0.044 D	0.30 D	0.20 +/- 0.014 D	0.36 D	0.22 +/- 0.012 D	0.20 +/- 0.029 D	0.31 D	0.19 +/- 0.000 D	0.28 D	0.19 +/- 0.023 D	0.22 +/- 0.054 D	0.31 D
22	160-176	GIGYAICIIAFYIASYY	16 D	7.72 D	0.56 +/- 0.011 D	0.60 D	0.58 +/- 0.038 D	0.56 +/- 0.044 D	0.64 D	0.59 +/- 0.055 D	0.63 D	0.60 +/- 0.011 D	0.59 +/- 0.032 D	0.68 D	0.62 +/- 0.018 D	0.62 D	0.56 +/- 0.023 D	0.61 +/- 0.054 D	0.66 D
23	167-182	IIAFYIASYYNTIMAW	15 D	7.84 D	0.61 +/- 0.006 D	0.62 D	0.59 +/- 0.033 D	0.63 +/- 0.042 D	0.58 D	0.66 +/- 0.003 D	0.69 D	0.60 +/- 0.018 D	0.61 +/- 0.074 D	0.75 D	0.65 +/- 0.033 D	0.88 D	0.59 +/- 0.027 D	0.67 +/- 0.056 D	0.61 D
24	175-191	YYNTIMAWALYYLISSF	16 D	D	-0.01 +/- 0.008 D	-0.08 D	-0.06 +/- 0.022 D	-0.01 +/- 0.022 D	-0.05 D	0.04 +/- 0.021 D	-0.03 D	0.02 +/- 0.037 D	-0.02 +/- 0.033 D	-0.01 D	0.00 +/- 0.015 D	-0.02 D	-0.02 +/- 0.017 D	-0.01 +/- 0.046 D	-0.03 D
25	175-192	YYNTIMAWALYYLISSFT	17 D	9.55 D	0.66 +/- 0.042 D	0.63 D	0.65 +/- 0.031 D	0.65 +/- 0.027 D	0.74 D	0.74 +/- 0.026 D	0.75 D	0.68 +/- 0.020 D	0.69 +/- 0.034 D	0.78 D	0.72 +/- 0.003 D	0.76 D	0.68 +/- 0.023 D	0.71 +/- 0.034 D	0.80 D
26	181-189	AWALYYLIS	8 D	3.25 D	1.98 +/- 0.030 D	2.06 D	2.14 +/- 0.044 D	2.44 +/- 0.014 D	2.57 D	1.93 +/- 0.022 D	1.96 D	2.05 +/- 0.038 D	2.31 +/- 0.033 D	2.44 D	1.96 +/- 0.057 D	2.14 D	2.07 +/- 0.015 D	2.44 +/- 0.040 D	2.62 D
27	188-203	ISSFTDQLPWTSCKNS	14 D	9.34 D	0.43 +/- 0.015 D	0.40 D	0.44 +/- 0.016 D	0.37 +/- 0.029 D	0.44 D	0.42 +/- 0.012 D	0.41 D	0.40 +/- 0.029 D	0.37 +/- 0.043 D	0.40 D	0.42 +/- 0.031 D	0.45 D	0.42 +/- 0.011 D	0.40 +/- 0.033 D	0.49 D
28	217-235	NITWTLHSTSPAEEFYTRH	17 D	9.82 D	8.70 +/- 0.035 D	8.49 D	8.69 +/- 0.043 D	8.39 +/- 0.194 D	8.62 D	8.64 +/- 0.004 D	8.67 D	8.53 +/- 0.113 D	8.50 +/- 0.034 D	8.55 D	8.69 +/- 0.042 D	8.61 D	8.63 +/- 0.187 D	8.70 +/- 0.049 D	8.24 D
29	224-230	STSPAEE	5 D	2.34 D	0.77 +/- 0.000 D	0.93 D	1.03 +/- 0.061 D	1.03 +/- 0.064 D	1.14 D	0.83 +/- 0.041 D	0.79 D	1.05 +/- 0.019 D	1.01 +/- 0.052 D	1.11 D	0.82 +/- 0.041 D	0.89 D	1.05 +/- 0.032 D	0.99 +/- 0.016 D	0.97 D
30	242-255	SKGLQDLGGISWQL	13 D	6.95 D	5.81 +/- 0.078 D	6.61 D	6.31 +/- 0.056 D	6.29 +/- 0.061 D	6.34 D	5.95 +/- 0.065 D	6.37 D	6.40 +/- 0.039 D	6.31 +/- 0.059 D	6.43 D	5.91 +/- 0.094 D	6.30 D	6.34 +/- 0.023 D	6.39 +/- 0.049 D	6.37 D
31	296-305	LVRGATLPGA	8 D	3.81 D	0.47 +/- 0.000 D	0.48 D	0.41 +/- 0.009 D	0.58 +/- 0.037 D	1.11 D	0.61 +/- 0.063 D	0.61 D	0.73 +/- 0.026 D	0.84 +/- 0.036 D	1.79 D	0.45 +/- 0.015 D	0.52 D	0.46 +/- 0.041 D	0.60 +/- 0.045 D	1.14 D
32	306-311	WRGVLF	5 D	2.60 D	0.03 +/- 0.006 D	0.11 D	0.17 +/- 0.017 D	0.56 +/- 0.034 D	1.36 D	0.15 +/- 0.015 D	0.24 D	0.42 +/- 0.007 D	0.88 +/- 0.035 D	1.41 D	0.03 +/- 0.015 D	0.14 D	0.39 +/- 0.007 D	1.00 +/- 0.044 D	1.09 D
33	312-320	YLKPNWQKL	7 D	3.87 D	1.07 +/- 0.012 D	1.45 D	1.93 +/- 0.067 D	2.33 +/- 0.072 D	2.76 D	1.74 +/- 0.131 D	2.15 D	2.46 +/- 0.171 D	3.18 +/- 0.041 D	3.43 D	1.69 +/- 0.078 D	1.85 D	2.24 +/- 0.130 D	2.44 +/- 0.037 D	2.94 D
34	319-329	KLLETGVWIDA	10 D	4.41 D	2.65 +/- 0.034 D	2.81 D	3.34 +/- 0.131 D	3.56 +/- 0.189 D	4.10 D	2.52 +/- 0.058 D	2.68 D	3.12 +/- 0.049 D	3.13 +/- 0.046 D	3.68 D	2.63 +/- 0.025 D	3.22 D	3.42 +/- 0.105 D	3.62 +/- 0.140 D	4.13 D
35	321-326	LETGVW	5 D	2.66 D	1.10 +/- 0.077 D	1.09 D	1.45 +/- 0.036 D	1.63 +/- 0.029 D	1.57 D	1.09 +/- 0.051 D	1.29 D	1.62 +/- 0.086 D	1.69 +/- 0.053 D	1.67 D	1.19 +/- 0.048 D	1.27 D	1.55 +/- 0.070 D	1.60 +/- 0.041 D	1.56 D
36	331-350	AQIFFSLGPGFGVLLAFASY	18 D	9.15 D	0.56 +/- 0.001 D	0.64 D	0.53 +/- 0.030 D	0.53 +/- 0.036 D	0.66 D	0.60 +/- 0.034 D	0.76 D	0.57 +/- 0.020 D	0.60 +/- 0.023 D	0.70 D	0.61 +/- 0.007 D	0.67 D	0.56 +/- 0.042 D	0.61 +/- 0.083 D	0.71 D
37	340-349	GFGVLLAFAS	9 D	3.35 D	2.77 +/- 0.030 D	2.77 D	2.84 +/- 0.049 D	2.75 +/- 0.050 D	2.79 D	2.81 +/- 0.046 D	2.81 D	2.87 +/- 0.033 D	2.75 +/- 0.041 D	2.81 D	2.78 +/- 0.024 D	2.77 D	2.84 +/- 0.057 D	2.78 +/- 0.043 D	2.79 D
38	354-364	NNNCYQDALVT	10 D	5.49 D	0.35 +/- 0.030 D	0.35 D	0.29 +/- 0.018 D	0.34 +/- 0.029 D	0.41 D	0.30 +/- 0.004 D	0.43 D	0.31 +/- 0.025 D	0.32 +/- 0.025 D	0.34 D	0.35 +/- 0.030 D	0.45 D	0.31 +/- 0.032 D	0.38 +/- 0.016 D	0.37 D
39	354-366	NNNCYQDALVTSV	12 D	6.21 D	0.42 +/- 0.009 D	0.46 D	0.34 +/- 0.024 D	0.36 +/- 0.046 D	0.52 D	0.43 +/- 0.004 D	0.61 D	0.40 +/- 0.042 D	0.44 +/- 0.053 D	0.57 D	0.43 +/- 0.024 D	0.60 D	0.33 +/- 0.026 D	0.44 +/- 0.064 D	0.54 D
40	383-398	LGYMAEMRNEDVSEVA	15 D	8.24 D	0.47 +/- 0.104 D	0.45 D	0.43 +/- 0.107 D	0.45 +/- 0.132 D	0.51 D	0.47 +/- 0.087 D	0.51 D	0.46 +/- 0.096 D	0.46 +/- 0.112 D	0.49 D	0.48 +/- 0.101 D	0.52 D	0.41 +/- 0.084 D	0.44 +/- 0.115 D	0.50 D
41	389-396	MRNEDVSE	7 D	D	0.30 +/- 0.069 D	0.31 D	0.32 +/- 0.084 D	0.34 +/- 0.082 D	0.45 D	0.38 +/- 0.080 D	0.29 D	0.31 +/- 0.068 D	0.36 +/- 0.091 D	0.31 D	0.31 +/- 0.086 D	0.32 D	0.33 +/- 0.103 D	0.40 +/- 0.076 D	0.31 D
42	397-406	VAKDAGPSLL	8 D	4.05 D	2.18 +/- 0.120 D	2.00 D	2.83 +/- 0.087 D	2.66 +/- 0.147 D	3.00 D	1.01 +/- 0.081 D	1.34 D	1.86 +/- 0.157 D	1.90 +/- 0.064 D	1.97 D	1.95 +/- 0.186 D	N/A	2.89 +/- 0.089 D	2.97 +/- 0.117 D	3.05 D
43	400-406	DAGPSLL	5 D	2.25 D	1.25 +/- 0.034 D	1.17 D	1.65 +/- 0.050 D	1.72 +/- 0.102 D	1.50 D	0.62 +/- 0.054 D	0.66 D	0.79 +/- 0.051 D	1.10 +/- 0.009 D	1.34 D	0.99 +/- 0.019 D	1.2 D	1.63 +/- 0.070 D	1.71 +/- 0.097 D	1.62 D
44	406-421	LFITYAEAIANMPAST	14 D	6.90 D	0.53 +/- 0.040 D	0.46 D	0.52 +/- 0.023 D	0.49 +/- 0.034 D	0.59 D	0.50 +/- 0.042 D	0.59 D	0.51 +/- 0.026 D	0.49 +/- 0.029 D	0.57 D	0.50 +/- 0.028 D	0.53 D	0.50 +/- 0.025 D	0.50 +/- 0.028 D	0.51 D
45	408-420	ITYAEAIANMPAS	11 D	7.54 D	0.42 +/- 0.007 D	0.33 D	0.40 +/- 0.047 D	0.38 +/- 0.069 D	0.41 D	0.49 +/- 0.000 D	0.49 D	0.41 +/- 0.037 D	0.42 +/- 0.019 D	0.40 D	0.39 +/- 0.059 D	0.5 D	0.43 +/- 0.023 D	0.42 +/- 0.026 D	0.41 D
46	408-422	ITYAEAIANMPASTF	13 D	6.23 D	0.55 +/- 0.013 D	0.42 D	0.47 +/- 0.081 D	0.45 +/- 0.052 D	0.51 D	0.36 +/- 0.040 D	0.24 D	0.27 +/- 0.058 D	0.29 +/- 0.025 D	0.37 D	0.55 +/- 0.038 D	0.48 D	0.45 +/- 0.049 D	0.54 +/- 0.103 D	0.49 D
47	483-499	FGGAYVVKLLEEYATGP	15 D	7.60 D	4.46 +/- 0.042 D	4.54 D	5.77 +/- 0.059 D	6.49 +/- 0.083 D	6.56 D	4.76 +/- 0.089 D	4.94 D	5.85 +/- 0.119 D	6.60 +/- 0.040 D	6.58 D	4.48 +/- 0.022 D	4.51 D	5.97 +/- 0.025 D	6.60 +/- 0.086 D	6.56 D
48	509-517	AVAVSWFYG	8 D	3.44 D	0.20 +/- 0.015 D	0.14 D	0.15 +/- 0.024 D	0.16 +/- 0.025 D	0.19 D	0.18 +/- 0.011 D	0.19 D	0.15 +/- 0.016 D	0.16 +/- 0.045 D	0.21 D	0.17 +/- 0.012 D	0.21 D	0.18 +/- 0.028 D	0.18 +/- 0.016 D	0.20 D
49	512-523	VSWFYGITQFCR	11 D	4.92 D	0.24 +/- 0.020 D	0.33 D	0.20 +/- 0.042 D	0.23 +/- 0.036 D	0.23 D	0.25 +/- 0.006 D	0.30 D	0.20 +/- 0.014 D	0.23 +/- 0.036 D	0.31 D	0.25 +/- 0.034 D	0.27 D	0.20 +/- 0.021 D	0.29 +/- 0.031 D	0.30 D
50	516-545	YGITQFCRDVKEMLGFSPGWFWRICWVAIS	28 D	D	1.21 +/- 0.004 D	1.06 D	1.18 +/- 0.031 D	1.19 +/- 0.052 D	1.20 D	1.29 +/- 0.034 D	1.29 D	1.26 +/- 0.025 D	1.26 +/- 0.053 D	1.28 D	1.31 +/- 0.091 D	1.27 D	1.27 +/- 0.063 D	1.27 +/- 0.063 D	1.24 D
51	519-548	TQFCRDVKEMLGFSPGWFWRICWVAISPLF	27 D	18.18 D	0.95 +/- 0.038 D	0.70 D	1.01 +/- 0.072 D	0.95 +/- 0.055 D	1.02 D	1.04 +/- 0.020 D	1.02 D	1.06 +/- 0.015 D	1.06 +/- 0.048 D	0.80 D	0.94 +/- 0.006 D	1.03 D	1.04 +/- 0.020 D	0.96 +/- 0.082 D	0.97 D
52	560-566	PPQLRLF	5 D	2.89 D	0.10 +/- 0.008 D	0.07 D	0.11 +/- 0.006 D	0.09 +/- 0.012 D	0.11 D	0.12 +/- 0.003 D	0.12 D	0.11 +/- 0.016 D	0.09 +/- 0.019 D	0.12 D	0.11 +/- 0.010 D	0.08 D	0.13 +/- 0.002 D	0.12 +/- 0.012 D	0.14 D
53	583-593	TSSFICIPTYI	9 D	4.50 D	0.19 +/- 0.094 D	0.16 D	0.17 +/- 0.099 D	0.16 +/- 0.096 D	0.14 D	0.18 +/- 0.114 D	0.20 D	0.20 +/- 0.106 D	0.20 +/- 0.101 D	0.17 D	0.19 +/- 0.098 D	0.18 D	0.16 +/- 0.101 D	0.19 +/- 0.099 D	0.18 D
54	590-595	PTYIAY	5 D	1.87 D	0.18 +/- 0.018 D	0.11 D	0.12 +/- 0.028 D	-0.03 +/- 0.126 D	0.15 D	0.20 +/- 0.008 D	0.18 D	0.13 +/- 0.022 D	0.16 +/- 0.015 D	0.19 D	0.17 +/- 0.000 D	0.14 D	0.12 +/- 0.010 D	0.15 +/- 0.017 D	0.14 D
55	595-607	YRLIITPGTFKER	11 D	5.99 D	1.55 +/- 0.007 D	1.89 D	2.40 +/- 0.111 D	3.05 +/- 0.041 D	3.71 D	1.62 +/- 0.066 D	1.95 D	2.47 +/- 0.081 D	3.34 +/- 0.125 D	4.59 D	1.49 +/- 0.025 D	1.92 D	2.34 +/- 0.088 D	3.14 +/- 0.117 D	3.41 D
56	598-607	IITPGTFKER	8 D	4.00 D	1.53 +/- 0.062 D	1.88 D	2.24 +/- 0.065 D	2.92 +/- 0.076 D	3.02 D	1.80 +/- 0.087 D	2.01 D	2.35 +/- 0.099 D	2.85 +/- 0.037 D	3.04 D	1.61 +/- 0.063 D	1.84 D	2.27 +/- 0.073 D	2.96 +/- 0.041 D	3.16 D
57	601-606	PGTFKE	5 D	2.52 D	1.82 +/- 0.008 D	1.86 D	1.87 +/- 0.043 D	1.84 +/- 0.016 D	1.76 D	1.87 +/- 0.001 D	1.76 D	1.91 +/- 0.028 D	1.86 +/- 0.033 D	1.74 D	1.85 +/- 0.017 D	1.82 D	1.87 +/- 0.035 D	1.86 +/- 0.025 D	1.83 D
58	608-619	IIKSITPETPTE	9 D	5.14 D	2.34 +/- 0.027 D	2.25 D	3.73 +/- 0.081 D	4.59 +/- 0.071 D	4.63 D	2.55 +/- 0.064 D	2.39 D	3.56 +/- 0.157 D	4.62 +/- 0.072 D	4.64 D	2.35 +/- 0.043 D	2.34 D	3.80 +/- 0.100 D	4.63 +/- 0.098 D	4.54 D
59	616-634	TPTEIPCGDIRLNAVLVPR	15 D	8.44 D	7.94 +/- 0.189 D	7.90 D	7.94 +/- 0.187 D	7.88 +/- 0.175 D	8.08 D	8.01 +/- 0.126 D	8.06 D	7.89 +/- 0.072 D	7.88 +/- 0.073 D	7.66 D	7.70 +/- 0.141 D	7.83 D	7.80 +/- 0.120 D	7.97 +/- 0.090 D	8.04 D
60	623-641	GDIRLNAVLVPRGSHHHHH	17 D	8.93 D	8.23 +/- 0.024 D	7.66 D	8.14 +/- 0.045 D	8.12 +/- 0.090 D	8.24 D	8.29 +/- 0.034 D	8.25 D	8.20 +/- 0.027 D	8.21 +/- 0.027 D	7.94 D	8.07 +/- 0.101 D	8.22 D	8.14 +/- 0.019 D	8.27 +/- 0.038 D	8.15 D
61	625-629	IRLNA	4 D	1.76 D	1.76 +/- 0.007 D	1.70 D	1.68 +/- 0.040 D	1.72 +/- 0.018 D	1.71 D	1.73 +/- 0.035 D	1.67 D	1.74 +/- 0.055 D	1.73 +/- 0.021 D	1.77 D	1.75 +/- 0.019 D	1.74 D	1.70 +/- 0.048 D	1.75 +/- 0.028 D	1.71 D
62	631-643	LVPRGSHHHHHHH	11 D	4.79 D	0.52 +/- 0.027 D	0.79 D	1.88 +/- 0.068 D	2.60 +/- 0.080 D	3.45 D	0.57 +/- 0.001 D	0.86 D	1.82 +/- 0.083 D	2.63 +/- 0.045 D	3.47 D	0.55 +/- 0.008 D	0.88 D	1.79 +/- 0.117 D	2.63 +/- 0.061 D	3.07 D

Supplementary Table 2. The relative deuterium uptake measured for Na+, ibogaine and cocaine states at all labelling time points (0.25 min – 480 min) for the 62 peptides identified from hSERT along with the theoretical maximum uptake and the measured maximum

Experiment: Na$^+$, K$^+$, 5-HT					Relative deuterium uptake														
					Na$^+$ state					Ibogaine state					Cocaine state				
Peptide number	Residues	Sequence	Theoretical uptake	Maximum uptake	0.25	1 min	10 min	60 min	480 min	0.25 min	1 min	10 min	60 min	480 min	0.25 min	1 min	10 min	60 min	480 min
1	1-14	METTPLNSQKQLSA	12 D	8.92 +/- 0.057 D	0.29 +/- 0.012 D	0.32 D	0.29 D	0.18 +/- 0.188 D	0.27 D	0.28 +/- 0.0003 D	0.39 D	0.35 D	0.30 0.040 D	0.28 D	0.31 +/- 0.066 D	0.36 D	0.36 D	0.26 +/- 0.100 D	0.23 D
2	5-12	PLNSQKQL	7 D	4.08 +/- 0.148 D	3.52 +/- 0.073 D	3.75 D	3.64 D	3.45 +/- 0.182 D	3.57 D	3.46 +/- 0.051 D	3.52 D	3.59 D	3.45 0.201 D	3.38 D	3.46 +/- 0.132 D	3.66 D	3.59 D	3.44 +/- 0.141 D	3.38 D
3	13-21	SACEDGEDC	8 D	3.32 +/- N/A D	2.73 +/- 0.034 D	2.70 D	2.64 D	2.46 +/- 0.247 D	2.60 D	2.69 +/- 0.055 D	2.62 D	2.58 D	2.57 0.002 D	2.56 D	2.68 +/- 0.048 D	2.67 D	2.62 D	2.58 +/- 0.008 D	2.51 D
4	13-28	SACEDGEDCQENGVLQ	15 D	6.47 +/- 0.151 D	6.02 +/- 0.065 D	6.27 D	5.83 D	5.74 +/- 0.110 D	5.83 D	6.00 +/- 0.178 D	6.04 D	5.90 D	5.78 0.310 D	5.95 D	5.98 +/- 0.116 D	6.00 D	5.84 D	5.82 +/- 0.208 D	6.00 D
5	22-28	QENGVLQ	6 D	2.84 +/- 0.048 D	2.54 +/- 0.009 D	2.55 D	2.49 D	2.42 +/- 0.060 D	2.51 D	2.51 +/- 0.002 D	2.47 D	2.43 D	2.45 0.002 D	2.46 D	2.49 +/- 0.041 D	2.53 D	2.48 D	2.42 +/- 0.025 D	2.48 D
6	29-37	KVVPTPGDK	6 D	2.51 +/- 0.350 D	2.23 +/- 0.059 D	2.38 D	2.21 D	2.02 +/- 0.120 D	2.17 D	2.19 +/- 0.095 D	2.21 D	2.10 D	2.10 0.052 D	2.18 D	2.15 +/- 0.064 D	2.27 D	2.19 D	2.06 +/- 0.056 D	2.14 D
7	29-47	KVVPTPGDKVESGQISNGY	16 D	8.72 +/- 0.147 D	7.98 +/- 0.027 D	8.09 D	7.99 D	7.77 +/- 0.287 D	7.89 D	7.98 +/- 0.002 D	7.87 D	7.82 D	7.71 0.301 D	7.90 D	7.97 +/- 0.038 D	7.95 D	7.89 D	7.82 +/- 0.321 D	8.06 D
8	30-46	VVPTPGDKVESGQISNG	14 D	7.64 +/- 0.090 D	7.12 +/- 0.104 D	7.04 D	7.04 D	6.70 +/- 0.250 D	6.68 D	7.08 +/- 0.040 D	7.13 D	6.93 D	6.85 0.058 D	6.86 D	7.11 +/- 0.148 D	7.12 D	7.02 D	6.95 +/- 0.080 D	6.99 D
9	38-47	VESGQISNGY	9 D	4.67 +/- 0.033 D	4.13 +/- 0.015 D	4.20 D	4.03 D	4.00 +/- 0.100 D	3.94 D	4.04 +/- 0.028 D	4.00 D	4.02 D	3.95 0.048 D	3.95 D	4.06 +/- 0.011 D	4.03 D	4.00 D	3.95 +/- 0.064 D	4.04 D
10	48-70	SAVPSPGAGDDTRHSIPATTTTL	19 D	9.22 +/- 0.146 D	8.07 +/- 0.035 D	8.11 D	8.16 D	7.90 +/- 0.422 D	8.13 D	8.12 +/- 0.102 D	8.07 D	8.17 D	7.94 0.529 D	8.22 D	8.19 +/- 0.229 D	8.30 D	8.25 D	8.06 +/- 0.518 D	8.34 D
11	70-88	LVAELHQGERETWGKKVDF	18 D	6.89 +/- 0.345 D	2.86 +/- 0.026 D	3.94 D	4.85 D	4.96 +/- 0.014 D	5.36 D	4.18 +/- 0.038 D	5.09 D	5.74 D	5.32 0.553 D	5.28 D	2.76 +/- 0.064 D	3.38 D	4.08 D	4.51 +/- 0.269 D	4.79 D
12	71-87	VAELHQGERETWGKKVD	16 D	6.83 +/- 0.264 D	3.32 +/- 0.187 D	4.50 D	5.40 D	5.42 +/- 0.729 D	5.78 D	4.96 +/- 0.332 D	5.25 D	5.34 D	5.34 0.639 D	5.76 D	3.15 +/- 0.232 D	3.92 D	4.73 D	5.15 +/- 0.465 D	5.70 D
13	71-88	VAELHQGERETWGKKVDF	17 D	6.79 +/- 0.291 D	3.10 +/- 0.156 D	4.42 D	5.48 D	5.46 +/- 0.483 D	5.63 D	4.71 +/- 0.514 D	5.21 D	5.35 D	5.47 0.510 D	5.38 D	2.96 +/- 0.186 D	3.74 D	4.28 D	5.09 +/- 0.566 D	5.60 D
14	78-89	ERETWGKKVDFL	11 D	4.49 +/- 0.201 D	1.14 +/- 0.106 D	1.94 D	2.66 D	2.75 +/- 0.068 D	3.22 D	2.14 +/- 0.023 D	2.67 D	3.15 D	3.03 0.359 D	3.07 D	1.10 +/- 0.085 D	1.50 D	1.97 D	2.25 +/- 0.024 D	2.69 D
15	89-94	LLSVIG	5 D	2.53 +/- 0.036 D	0.03 +/- 0.024 D	0.13 D	0.42 D	1.24 +/- 0.035 D	1.96 D	0.20 +/- 0.002 D	0.62 D	1.54 D	2.08 0.038 D	2.23 D	0.00 +/- 0.004 D	0.06 D	0.12 D	0.22 +/- 0.037 D	1.04 D
16	89-95	LLSVIGY	6 D	3.12 +/- 0.025 D	0.08 +/- 0.007 D	0.21 D	0.62 D	1.57 +/- 0.130 D	2.64 D	0.32 +/- 0.004 D	0.74 D	1.82 D	2.55 0.256 D	2.77 D	0.08 +/- 0.001 D	0.16 D	0.24 D	0.28 +/- 0.046 D	N/A
17	99-109	LGNVWRFPYIC	9 D	4.91 +/- 0.061 D	0.65 +/- 0.021 D	0.81 D	0.92 D	1.38 +/- 0.300 D	3.20 D	0.25 +/- 0.005 D	0.28 D	0.66 D	1.34 0.022 D	2.07 D	0.65 +/- 0.072 D	0.90 D	1.06 D	1.66 +/- 0.159 D	3.19 D
18	126-150	IFGGIPLFYMELALGQYHRNGCISI	23 D	7.67 +/- 0.540 D	4.20 +/- 0.214 D	5.40 D	6.80 D	6.56 +/- 0.432 D	7.36 D	4.27 +/- 0.580 D	5.40 D	6.71 D	6.47 0.444 D	7.26 D	4.37 +/- 0.433 D	5.72 D	6.77 D	6.66 +/- 0.329 D	7.29 D
19	138-150	ALGQYHRNGCISI	12 D	5.38 +/- 0.167 D	4.88 +/- 0.018 D	5.07 D	4.57 D	4.72 +/- 0.252 D	4.76 D	4.85 +/- 0.112 D	4.80 D	4.52 D	4.71 0.300 D	4.96 D	4.76 +/- 0.000 D	4.65 D	4.69 D	4.78 +/- 0.224 D	4.84 D
20	140-158	GQYHRNGCISIWRKICPIF	17 D	5.37 +/- 0.157 D	4.90 +/- 0.061 D	5.03 D	4.95 D	4.83 +/- 0.238 D	4.83 D	4.92 +/- 0.070 D	4.88 D	4.91 D	4.75 0.194 D	4.99 D	4.91 +/- 0.150 D	4.83 D	4.82 D	4.89 +/- 0.230 D	4.93 D
21	145-153	NGCISIWRK	8 D	3.73 +/- 0.110 D	0.00 +/- 0.174 D	0.11 D	0.06 D	-0.11 +/- 0.078 D	0.02 D	0.02 +/- 0.159 D	0.16 D	0.16 D	0.00 0.012 D	0.06 D	0.04 +/- 0.158 D	0.17 D	0.19 D	-0.04 +/- 0.029 D	-0.03 D
22	160-176	GIGYAICIIAFYIASYY	16 D	7.65 +/- 0.156 D	0.44 +/- 0.022 D	0.51 D	0.47 D	0.29 +/- 0.243 D	0.45 D	0.43 +/- 0.007 D	0.55 D	0.55 D	0.48 0.024 D	0.45 D	0.52 +/- 0.079 D	0.64 D	0.54 D	0.41 +/- 0.091 D	0.37 D
23	167-182	IIAFYIASYYNTIMAW	15 D	8.18 +/- 0.211 D	0.43 +/- 0.024 D	0.57 D	0.43 D	0.28 +/- 0.289 D	0.46 D	0.41 +/- 0.043 D	0.58 D	0.53 D	0.47 0.022 D	0.41 D	0.51 +/- 0.028 D	0.64 D	0.60 D	0.41 +/- 0.103 D	0.31 D
24	175-191	YYNTIMAWALYYLISSF	16 D	N/A	N/A	N/A	N/A	N/A	N/A	N/A	N/A	N/A	N/A	N/A	N/A	N/A	N/A	N/A	N/A
25	175-192	YYNTIMAWALYYLISSFT	17 D	9.24 +/- 0.047 D	0.42 +/- 0.056 D	0.50 D	0.40 D	0.27 +/- 0.250 D	0.38 D	0.46 +/- 0.026 D	0.59 D	0.57 D	0.47 0.018 D	0.46 D	0.51 +/- 0.140	0.63 D	0.57	0.44 +/- 0.123 D	0.31 D
26	181-189	AWALYYLIS	8 D	3.53 +/- 0.130 D	1.99 +/- 0.079 D	2.27 D	2.28 D	2.39 +/- 0.061 D	2.89 D	1.90 +/- 0.327 D	2.28 D	2.31 D	2.42 0.388 D	2.91 D	1.94 +/- 0.153	2.10 D	2.26	2.44 +/- 0.247 D	2.94 D
27	188-203	ISSFTDQLPWTSCKNS	14 D	9.20 +/- 0.064 D	0.26 +/- 0.001 D	0.27 D	0.25 D	0.16 +/- 0.129 D	0.23 D	0.25 +/- 0.051 D	0.27 D	0.32 D	0.20 0.038 D	0.19 D	0.29 +/- 0.019	0.36 D	0.33	0.18 +/- 0.117 D	0.21 D
28	217-235	NITWTLHSTSPAEEFYTRH	17 D	9.29 +/- 0.050 D	8.40 +/- 0.076 D	8.28 D	8.46 D	8.19 +/- 0.294 D	8.21 D	8.37 +/- 0.046 D	8.36 D	8.30 D	8.27 0.355 D	8.34 D	8.46 +/- 0.079	8.40 D	8.43	8.37 +/- 0.152 D	8.41 D
29	224-230	STSPAEE	5 D	2.29 +/- 0.049 D	0.76 +/- 0.015 D	1.03 D	1.07 D	1.05 +/- 0.228 D	1.18 D	0.80 +/- 0.016 D	1.10 D	1.03 D	1.16 0.003 D	1.02 D	0.71 +/- 0.037	1.00 D	1.06	1.09 +/- 0.047 D	0.92 D
30	242-255	SKGLQDLGGISWQL	13 D	6.71 +/- 0.160 D	5.63 +/- 0.005 D	6.47 D	6.26 D	6.15 +/- 0.319 D	6.31 D	5.67 +/- 0.025 D	6.24 D	6.30 D	5.98 0.256 D	6.23 D	5.64 +/- 0.114	6.34 D	6.32	6.12 +/- 0.362 D	6.24 D
31	296-305	LVRGATLPGA	8 D	3.63 +/- 0.078 D	0.42 +/- 0.036 D	0.45 D	0.38 D	0.40 +/- 0.151 D	0.77 D	0.37 +/- 0.049 D	0.42 D	0.45 D	0.52 0.013 D	0.97 D	0.46 +/- 0.111	0.43 D	0.45	0.40 +/- 0.051 D	0.61 D
32	306-311	WRGVLF	5 D	2.40 +/- 0.078 D	0.07 +/- 0.009 D	0.07 D	0.10 D	0.42 +/- 0.119 D	1.31 D	0.06 +/- 0.012 D	0.08 D	0.20 D	0.81 0.044 D	1.13 D	0.07 +/- 0.001	0.13 D	0.14	0.40 +/- 0.022 D	1.05 D
33	312-320	YLKPNWQKL	7 D	4.03 +/- 0.079 D	0.84 +/- 0.013 D	1.39 D	1.76 D	2.00 +/- 0.232 D	2.32 D	1.24 +/- 0.032 D	1.76 D	2.29 D	2.53 0.185 D	2.81 D	0.79 +/- 0.021	1.37 D	1.70	1.89 +/- 0.030 D	2.14 D
34	319-329	KLLETGVWIDA	10 D	4.62 +/- 0.215 D	2.75 +/- 0.102 D	3.01 D	3.28 D	3.28 +/- 0.206 D	3.86 D	2.67 +/- 0.314 D	3.08 D	3.41 D	3.57 0.170 D	4.17 D	2.65 +/- 0.207	2.81 D	3.28	3.58 +/- 0.169 D	4.18 D
35	321-326	LETGVW	5 D	2.60 +/- 0.036 D	1.03 +/- 0.047 D	1.15 D	1.26 D	1.43 +/- 0.120 D	1.56 D	1.05 +/- 0.006 D	1.19 D	1.45 D	1.56 0.116 D	1.44 D	1.02 +/- 0.011	1.02 D	1.23	1.48 +/- 0.006 D	1.41 D
36	331-350	AQIFFSLGPGFGVLLAFASY	18 D	8.95 +/- 0.073 D	0.47 +/- 0.000 D	0.54 D	0.62 D	0.40 +/- 0.382 D	0.56 D	0.46 +/- 0.030 D	0.90 D	0.59 D	0.49 0.016 D	0.48 D	0.52 +/- 0.029	0.62 D	0.62	0.49 +/- 0.162 D	0.34 D
37	340-349	GFGVLLAFAS	9 D	3.21 +/- 0.114 D	2.85 +/- 0.074 D	2.92 D	2.82 D	2.76 +/- 0.222 D	2.83 D	2.83 +/- 0.104 D	2.93 D	2.87 D	2.73 0.205 D	2.97 D	2.85 +/- 0.151	2.89 D	2.91	2.75 +/- 0.201 D	3.01 D
38	354-364	NNNCYQDALVT	10 D	5.62 +/- 0.064 D	0.09 +/- 0.139 D	0.21 D	0.09 D	-0.05 +/- 0.166 D	0.10 D	0.07 +/- 0.123 D	0.13 D	0.13 D	0.08 0.006 D	0.01 D	0.12 +/- 0.189	0.26 D	0.21	-0.01 +/- 0.092 D	-0.07 D
39	354-366	NNNCYQDALVTSV	12 D	6.27 +/- 0.056 D	-0.18 +/- 0.297 D	0.00 D	-0.06 D	-0.44 +/- 0.231 D	-0.26 D	-0.14 +/- 0.346 D	-0.01 D	-0.01 D	-0.31 0.001 D	-0.34 D	-0.07 +/- 0.438	0.07 D	0.00	-0.37 +/- 0.090 D	-0.47 D
40	383-398	LGYMAEMRNEDVSEVA	15 D	8.52 +/- 0.047 D	0.21 +/- 0.047 D	0.27 D	0.19 D	0.10 +/- 0.221 D	0.09 D	0.20 +/- 0.028 D	0.32 D	0.29 D	0.18 0.079 D	0.15 D	0.24 +/- 0.102	0.31 D	0.30	0.11 +/- 0.093 D	0.02 D
41	389-396	MRNEDVSE	7 D	N/A	0.26 +/- 0.052 D	0.24 D	0.19 D	0.19 +/- 0.088 D	0.34 D	0.25 +/- 0.056 D	0.27 D	0.28 D	0.30 0.065 D	0.30 D	0.25 +/- 0.078	0.28 D	0.28	0.24 +/- 0.012 D	0.27 D
42	397-406	VAKDAGPSLL	8 D	3.80 +/- 0.108 D	2.35 +/- 0.047 D	2.61 D	2.52 D	2.49 +/- 0.052 D	2.61 D	1.38 +/- 0.047 D	1.52 D	2.08 D	2.54 0.161 D	2.57 D	2.30 +/- 0.119	2.53 D	2.66	2.54 +/- 0.054 D	2.58 D
43	400-406	DAGPSLL	5 D	2.33 +/- 0.030 D	1.36 +/- 0.053 D	1.54 D	1.58 D	1.60 +/- 0.147 D	1.61 D	0.65 +/- 0.012 D	0.75 D	1.33 D	1.72 0.058 D	1.52 D	1.27 +/- 0.013	1.48 D	1.67	1.68 +/- 0.025 D	1.68 D
44	406-421	LFITYAEAIANMPAST	14 D	6.78 +/- 0.101 D	0.43 +/- 0.010 D	0.46 D	0.43 D	0.29 +/- 0.163 D	0.43 D	0.43 +/- 0.028 D	0.51 D	0.51 D	0.44 0.009 D	0.44 D	0.45 +/- 0.064	0.55 D	0.51	0.38 +/- 0.044 D	0.33 D
45	408-420	ITYAEAIANMPAS	11 D	7.45 +/- 0.075 D	0.23 +/- 0.060 D	0.27 D	0.32 D	0.16 +/- 0.148 D	0.25 D	0.23 +/- 0.007 D	0.34 D	0.34 D	0.26 0.027 D	0.23 D	0.26 +/- 0.052	0.3 D	0.32	0.26 +/- 0.090 D	0.11 D
46	408-422	ITYAEAIANMPASTF	13 D	6.28 +/- 0.135 D	0.39 +/- 0.002 D	0.38 D	0.34 D	0.32 +/- 0.207 D	0.35 D	0.42 +/- 0.008 D	0.55 D	0.47 D	0.40 0.009 D	0.43 D	0.43 +/- 0.004	0.44 D	0.42	0.36 +/- 0.089 D	0.35 D
47	483-499	FGGAYVVKLLEEYATGP	15 D	7.53 +/- 0.103 D	4.71 +/- 0.173 D	5.06 D	6.01 D	6.63 +/- 0.296 D	6.85 D	4.72 +/- 0.020 D	4.81 D	5.77 D	6.50 0.313 D	6.69 D	4.70 +/- 0.077	4.98 D	5.95	6.69 +/- 0.206 D	6.73 D
48	509-517	AVAVSWFYG	8 D	3.14 +/- 0.077 D	0.06 +/- 0.047 D	0.17 D	0.07 D	0.03 +/- 0.030 D	0.09 D	0.07 +/- 0.030 D	0.09 D	0.07 D	0.06 0.021 D	0.05 D	0.08 +/- 0.034	0.1 D	0.06	0.05 +/- 0.006 D	0.01 D
49	512-523	VSWFYGITQFCR	11 D	4.94 +/- 0.217 D	0.00 +/- 0.120 D	0.17 D	0.03 D	-0.13 +/- 0.120 D	0.02 D	-0.04 +/- 0.062 D	0.11 D	0.10 D	-0.05 0.073 D	-0.10 D	0.04 +/- 0.116	0.18 D	0.08	-0.08 +/- 0.034 D	-0.20 D
50	516-545	YGITQFCRDVKEMLGFSPGWFWRICWVAIS	28 D	N/A	0.86 +/- 0.045 D	0.85 D	0.79 D	0.67 +/- 0.396 D	0.88 D	0.86 +/- 0.134 D	1.06 D	1.06 D	0.93 0.035 D	0.91 D	0.95 +/- 0.043	1.1 D	1.03	0.86 +/- 0.190 D	0.73 D
51	519-548	TQFCRDVKEMLGFSPGWFWRICWVAISPLF	27 D	18.29 +/- 0.124 D	0.68 +/- 0.098 D	0.67 D	0.73 D	0.63 +/- 0.214 D	0.75 D	0.75 +/- 0.122 D	0.90 D	0.91 D	0.80 0.002 D	0.80 D	0.78 +/- 0.018	0.92 D	0.89	0.79 +/- 0.090 D	0.68 D
52	560-566	PPQLRLF	5 D	2.78 +/- 0.012 D	0.01 +/- 0.045 D	0.06 D	0.01 D	-0.02 +/- 0.026 D	0.04 D	0.01 +/- 0.017 D	0.06 D	0.05 D	0.03 0.023 D	0.02 D	0.03 +/- 0.052	0.1 D	0.06	0.01 +/- 0.013 D	-0.01 D
53	583-593	TSSFICIPTYI	9 D	4.34 +/- 0.229 D	0.16 +/- 0.066 D	0.14 D	0.04 D	-0.04 +/- 0.246 D	-0.01 D	0.13 +/- 0.056 D	0.13 D	0.09 D	0.10 0.115 D	-0.05 D	0.21 +/- 0.000	0.21 D	0.14	0.00 +/- 0.106 D	-0.09 D
54	590-595	PTYIAY	5 D	2.19 +/- 0.031 D	-0.06 +/- 0.156 D	0.02 D	0.12 D	-0.03 +/- 0.088 D	0.09 D	0.01 +/- 0.008 D	0.01 D	0.10 D	0.05 0.013 D	-0.02 D	0.04 +/- 0.009	0.04 D	0.02	0.03 +/- 0.007 D	-0.05 D
55	595-607	YRLIITPGTFKER	11 D	6.30 +/- 0.085 D	1.71 +/- 0.020 D	2.24 D	2.47 D	3.16 +/- 0.210 D	3.84 D	1.75 +/- 0.008 D	2.21 D	2.46 D	3.12 0.013 D	3.99 D	1.71 +/- 0.026	2.21 D	2.46	3.20 +/- 0.081 D	3.95 D
56	598-607	IITPGTFKER	8 D	4.05 +/- 0.090 D	1.32 +/- 0.039 D	1.88 D	2.07 D	2.73 +/- 0.091 D	3.55 D	1.38 +/- 0.079 D	1.79 D	1.96 D	2.62 0.032 D	3.47 D	1.33 +/- 0.032	1.85 D	2.02	2.75 +/- 0.117 D	3.54 D
57	601-606	PGTFKE	5 D	2.44 +/- 0.029 D	1.97 +/- 0.009 D	1.95 D	1.92 D	1.90 +/- 0.042 D	1.90 D	1.95 +/- 0.039 D	1.92 D	1.94 D	1.91 0.070 D	1.87 D	1.93 +/- 0.036	1.94 D	1.93	1.92 +/- 0.014 D	1.92 D
58	608-619	IIKSITPETPTE	9 D	5.20 +/- 0.083 D	2.36 +/- 0.024 D	2.54 D	3.68 D	4.50 +/- 0.336 D	4.67 D	2.33 +/- 0.094 D	2.36 D	3.57 D	4.41 0.309 D	4.46 D	2.29 +/- 0.134	2.37 D	3.65	4.38 +/- 0.195 D	4.55 D
59	616-634	TPTEIPCGDIRLNAVLVPR	15 D	8.59 +/- 0.106 D	7.93 +/- 0.083 D	7.98 D	7.84 D	7.67 +/- 0.253 D	7.83 D	7.93 +/- 0.047 D	7.87 D	7.88 D	7.69 0.264 D	7.89 D	7.89 +/- 0.017	8.02 D	7.79	7.84 +/- 0.137 D	8.02 D
60	623-641	GDIRLNAVLVPRGSHHHHH	17 D	8.70 +/- 0.304 D	7.86 +/- 0.004 D	8.10 D	8.00 D	7.72 +/- 0.386 D	8.04 D	7.85 +/- 0.031 D	7.94 D	8.02 D	7.71 0.440 D	7.80 D	7.78 +/- 0.036	8.06 D	7.97	7.75 +/- 0.446 D	7.74 D
61	625-629	IRLNA	4 D	1.86 +/- 0.036 D	1.73 +/- 0.054 D	1.80 D	1.70 D	1.58 +/- 0.097 D	1.69 D	1.69 +/- 0.046 D	1.67 D	1.64 D	1.65 0.027 D	1.64 D	1.68 +/- 0.070	1.71 D	1.67	1.63 +/- 0.010 D	1.64 D
62	631-643	LVPRGSHHHHHHH	11 D	N/A	N/A	N/A	N/A	N/A	N/A	N/A	N/A	N/A	N/A	N/A	N/A	N/A	N/A	N/A	N/A

Supplementary Table 3. The relative deuterium uptake measured for Na+ and S-citalopram states at all labelling time points (0.25 min – 480 min) for the 62 peptides identified from hSERT along with the theoretical maximum uptake

Experiment: Na^+, S-Citalopram					Relative deuterium uptake									
					Na^+ state					S-Citalopram state				
Peptide number	Residues	Sequence	Theoretical uptake	Maximum uptake	0.25	1 min	10 min	60 min	480 min	0.25 min	1 min	10 min	60 min	480 min
1	1-14	METTPLNSQKQLSA	12 D	N/A	0.32 +/- 0.017 D	0.34 +/- 0.041 D	0.26 +/- 0.034 D	0.27 +/- 0.157 D	0.29 +/- 0.03 D	0.32 +/- 0.066 D	0.33 +/- 0.040 D	0.28 +/- 0.146 D	0.29 +/- 0.102 D	0.29 +/- 0.08 D
2	5-12	PLNSQKQL	7 D	N/A	3.83 +/- 0.222 D	3.80 +/- 0.190 D	3.89 +/- 0.363 D	3.91 +/- 0.247 D	3.89 +/- 0.464 D	3.93 +/- 0.314 D	3.77 +/- 0.190 D	3.87 +/- 0.448 D	3.85 +/- 0.305 D	3.82 +/- 0.526 D
3	13-21	SACEDGEDC	8 D	N/A	2.81 +/- 0.091 D	2.75 +/- 0.043 D	2.79 +/- 0.202 D	2.66 +/- 0.268 D	2.76 +/- 0.224 D	2.75 +/- 0.099 D	2.77 +/- 0.063 D	2.74 +/- 0.194 D	2.74 +/- 0.175 D	2.77 +/- 0.257 D
4	13-28	SACEDGEDCQENGVLQ	15 D	N/A	6.17 +/- 0.144 D	6.27 +/- 0.001 D	5.83 +/- N/A D	6.02 +/- 0.190 D	5.83 +/- N/A D	6.06 +/- 0.112 D	6.17 +/- 0.042 D	5.88 +/- N/A D	6.08 +/- 0.096 D	5.99 +/- N/A D
5	22-28	QENGVLQ	6 D	N/A	2.66 +/- 0.099 D	2.65 +/- 0.094 D	2.60 +/- 0.157 D	2.61 +/- 0.171 D	2.64 +/- 0.188 D	2.64 +/- 0.086 D	2.63 +/- 0.133 D	2.61 +/- 0.192 D	2.60 +/- 0.174 D	2.59 +/- 0.253 D
6	29-37	KVVPTPGDK	6 D	N/A	2.43 +/- 0.171 D	2.46 +/- 0.125 D	2.29 +/- 0.113 D	2.38 +/- 0.311 D	2.30 +/- 0.186 D	2.40 +/- 0.206 D	2.43 +/- 0.212 D	2.27 +/- 0.136 D	2.42 +/- 0.232 D	2.28 +/- 0.238 D
7	29-47	KVVPTPGDKVESGQISNGY	16 D	N/A	8.16 +/- 0.146 D	8.22 +/- 0.165 D	8.09 +/- 0.153 D	8.14 +/- 0.137 D	8.11 +/- 0.318 D	8.08 +/- 0.211 D	8.15 +/- 0.230 D	8.01 +/- 0.275 D	8.14 +/- 0.168 D	8.14 +/- 0.258 D
8	30-46	VVPTPGDKVESGQISNG	14 D	N/A	7.31 +/- 0.165 D	7.16 +/- 0.159 D	7.04 +/- N/A D	6.85 +/- 0.287 D	6.68 +/- N/A D	7.16 +/- 0.088 D	7.12 +/- 0.062 D	6.61 +/- N/A D	6.83 +/- 0.129 D	6.72 +/- N/A D
9	38-47	VESGQISNGY	9 D	N/A	3.90 +/- 0.224 D	3.93 +/- 0.294 D	3.85 +/- 0.256 D	3.89 +/- 0.164 D	3.83 +/- 0.158 D	3.88 +/- 0.201 D	3.85 +/- 0.243 D	3.84 +/- 0.282 D	3.88 +/- 0.161 D	3.87 +/- 0.204 D
10	48-70	SAVPSPGAGDDTRHSIPATTTTL	19 D	N/A	8.33 +/- 0.272 D	8.35 +/- 0.208 D	8.39 +/- 0.322 D	8.31 +/- 0.243 D	8.44 +/- 0.439 D	8.35 +/- 0.148 D	8.37 +/- 0.155 D	8.42 +/- 0.296 D	8.33 +/- 0.200 D	8.45 +/- 0.320 D
11	70-88	LVAELHQGERETWGKKVDF	18 D	N/A	3.05 +/- 0.240 D	3.88 +/- 0.084 D	4.85 +/- N/A D	5.55 +/- 0.524 D	5.36 +/- N/A D	2.99 +/- 0.301 D	3.71 +/- 0.159 D	4.14 +/- N/A D	4.87 +/- 0.297 D	5.21 +/- N/A D
12	71-87	VAELHQGERETWGKKVD	16 D	N/A	3.64 +/- 0.159 D	4.35 +/- 0.148 D	5.69 +/- 0.409 D	5.82 +/- 0.165 D	5.91 +/- 0.191 D	3.57 +/- 0.143 D	4.28 +/- 0.029 D	5.47 +/- 0.556 D	5.61 +/- 0.307 D	5.96 +/- 0.198 D
13	71-88	VAELHQGERETWGKKVDF	17 D	N/A	3.25 +/- 0.046 D	4.40 +/- 0.261 D	5.59 +/- 0.143 D	5.97 +/- 0.350 D	6.11 +/- 0.678 D	3.15 +/- 0.257 D	4.21 +/- 0.133 D	5.40 +/- 1.066 D	5.45 +/- 0.266 D	5.79 +/- 0.204 D
14	78-89	ERETWGKKVDFL	11 D	N/A	1.20 +/- 0.114 D	1.81 +/- 0.111 D	2.69 +/- 0.037 D	2.93 +/- 0.229 D	3.17 +/- 0.068 D	1.19 +/- 0.064 D	1.69 +/- 0.063 D	2.16 +/- 0.202 D	2.49 +/- 0.244 D	3.01 +/- 0.147 D
15	89-94	LLSVIG	5 D	N/A	0.05 +/- 0.020 D	0.08 +/- 0.039 D	0.42 +/- 0.001 D	1.31 +/- 0.102 D	2.00 +/- 0.050 D	0.02 +/- 0.027 D	0.04 +/- 0.030 D	0.10 +/- 0.033 D	0.36 +/- 0.152 D	1.51 +/- 0.143 D
16	89-95	LLSVIGY	6 D	N/A	0.08 +/- 0.020 D	0.15 +/- 0.061 D	0.59 +/- 0.041 D	1.62 +/- 0.149 D	2.62 +/- 0.021 D	0.07 +/- 0.046 D	0.10 +/- 0.036 D	0.20 +/- 0.011 D	0.43 +/- 0.159 D	1.91 +/- 0.271 D
17	99-109	LGNVWRFPYIC	9 D	N/A	0.75 +/- 0.210 D	1.00 +/- 0.242 D	1.20 +/- 0.407 D	1.63 +/- 0.372 D	3.47 +/- 0.387 D	0.65 +/- 0.213 D	0.85 +/- 0.294 D	1.02 +/- 0.553 D	1.31 +/- 0.325 D	2.50 +/- 0.638 D
18	126-150	IFGGIPLFYMELALGQYHRNGCISI	23 D	N/A	4.20 +/- 0.390 D	5.22 +/- 0.433 D	6.73 +/- 0.098 D	7.16 +/- 0.215 D	7.51 +/- 0.201 D	4.23 +/- 0.489 D	5.33 +/- 0.475 D	6.76 +/- 0.037 D	7.21 +/- 0.204 D	7.57 +/- 0.384 D
19	138-150	ALGQYHRNGCISI	12 D	N/A	5.89 +/- 0.866 D	5.97 +/- 0.796 D	5.39 +/- 1.160 D	5.99 +/- 0.733 D	5.58 +/- 1.156 D	5.89 +/- 0.885 D	5.90 +/- 0.845 D	5.44 +/- 1.130 D	6.00 +/- 0.762 D	5.60 +/- 1.077 D
20	140-158	GQYHRNGCISIWRKICPIF	17 D	N/A	4.93 +/- 0.011 D	5.03 +/- 0.007 D	4.95 +/- N/A D	4.88 +/- 0.117 D	4.83 +/- N/A D	4.91 +/- 0.085 D	4.99 +/- 0.019 D	4.92 +/- N/A D	4.88 +/- 0.104 D	4.94 +/- N/A D
21	145-153	NGCISIWRK	8 D	N/A	0.43 +/- 0.424 D	0.73 +/- 0.888 D	0.06 +/- N/A D	0.59 +/- 1.159 D	0.02 +/- N/A D	0.47 +/- 0.435 D	0.75 +/- 0.794 D	-0.01 +/- N/A D	0.90 +/- 1.374 D	0.02 +/- N/A D
22	160-176	GIGYAICIIAFYIASYY	16 D	N/A	0.33 +/- 0.298 D	0.34 +/- 0.263 D	0.17 +/- 0.423 D	0.28 +/- 0.254 D	0.45 +/- N/A D	0.37 +/- 0.240 D	0.39 +/- 0.245 D	0.13 +/- 0.351 D	0.32 +/- 0.263 D	0.18 +/- 0.308 D
23	167-182	IIAFYIASYYNTIMAW	15 D	N/A	0.49 +/- 0.071 D	0.57 +/- 0.006 D	0.43 +/- N/A D	0.37 +/- 0.258 D	0.46 +/- N/A D	0.48 +/- 0.038 D	0.51 +/- 0.016 D	0.35 +/- N/A D	0.46 +/- 0.162 D	0.35 +/- N/A D
24	175-191	YYNTIMAWALYYLISSF	16 D	N/A	0.09 +/- 0.152 D	0.14 +/- 0.182 D	-0.01 +/- N/A D	0.11 +/- 0.109 D	0.05 +/- N/A D	0.09 +/- 0.081 D	0.09 +/- 0.086 D	-0.02 +/- N/A D	0.21 +/- 0.161 D	0.16 +/- N/A D
25	175-192	YYNTIMAWALYYLISSFT	17 D	N/A	0.46 +/- 0.125 D	0.47 +/- 0.039 D	0.38 +/- 0.036 D	0.37 +/- 0.193 D	0.44 +/- 0.082 D	0.47 +/- 0.059 D	0.48 +/- 0.134 D	0.36 +/- 0.077 D	0.41 +/- 0.094 D	0.37 +/- 0.028 D
26	181-189	AWALYYLIS	8 D	N/A	2.21 +/- 0.216 D	2.31 +/- 0.155 D	2.33 +/- 0.071 D	2.67 +/- 0.166 D	3.00 +/- 0.149 D	2.17 +/- 0.155 D	2.31 +/- 0.099 D	2.31 +/- 0.110 D	2.71 +/- 0.127 D	3.05 +/- 0.063 D
27	188-203	ISSFTDQLPWTSCKNS	14 D	N/A	0.32 +/- 0.067 D	0.32 +/- 0.052 D	0.32 +/- 0.098 D	0.28 +/- 0.147 D	0.29 +/- 0.075 D	0.34 +/- 0.053 D	0.31 +/- 0.068 D	0.20 +/- 0.075 D	0.28 +/- 0.119 D	0.31 +/- 0.184 D
28	217-235	NITWTLHSTSPAEEFYTRH	17 D	N/A	8.49 +/- 0.122 D	8.45 +/- 0.158 D	8.51 +/- 0.071 D	8.40 +/- 0.214 D	8.46 +/- 0.353 D	8.46 +/- 0.156 D	8.48 +/- 0.160 D	8.45 +/- 0.193 D	8.51 +/- 0.087 D	8.44 +/- 0.279 D
29	224-230	STSPAEE	5 D	N/A	0.81 +/- 0.073 D	1.16 +/- 0.134 D	1.17 +/- 0.146 D	1.21 +/- 0.235 D	1.37 +/- 0.256 D	0.83 +/- 0.064 D	1.21 +/- 0.122 D	1.20 +/- 0.064 D	1.26 +/- 0.163 D	1.36 +/- 0.325 D
30	242-255	SKGLQDLGGISWQL	13 D	N/A	5.76 +/- 0.124 D	6.38 +/- 0.090 D	6.32 +/- 0.074 D	6.38 +/- 0.153 D	6.43 +/- 0.167 D	5.73 +/- 0.088 D	6.27 +/- 0.059 D	6.38 +/- 0.221 D	6.36 +/- 0.125 D	6.38 +/- 0.264 D
31	296-305	LVRGATLPGA	8 D	N/A	0.46 +/- 0.037 D	0.44 +/- 0.022 D	0.42 +/- 0.061 D	0.46 +/- 0.116 D	0.81 +/- 0.056 D	0.45 +/- 0.042 D	0.43 +/- 0.042 D	0.39 +/- 0.032 D	0.43 +/- 0.067 D	0.72 +/- 0.118 D
32	306-311	WRGVLF	5 D	N/A	0.04 +/- 0.018 D	0.04 +/- 0.025 D	0.09 +/- 0.017 D	0.42 +/- 0.072 D	1.29 +/- 0.036 D	0.05 +/- 0.031 D	0.05 +/- 0.039 D	0.07 +/- 0.062 D	0.39 +/- 0.044 D	1.17 +/- 0.035 D
33	312-320	YLKPNWQKL	7 D	N/A	0.86 +/- 0.121 D	1.37 +/- 0.162 D	1.77 +/- 0.003 D	1.95 +/- 0.195 D	2.37 +/- 0.070 D	0.84 +/- 0.121 D	1.37 +/- 0.215 D	1.66 +/- 0.146 D	1.90 +/- 0.187 D	2.29 +/- 0.033 D
34	319-329	KLLETGVWIDA	10 D	N/A	2.99 +/- 0.403 D	3.05 +/- 0.372 D	3.42 +/- 0.196 D	3.70 +/- 0.386 D	4.18 +/- 0.452 D	2.87 +/- 0.338 D	3.02 +/- 0.348 D	3.45 +/- 0.207 D	3.80 +/- 0.244 D	4.27 +/- 0.162 D
35	321-326	LETGVW	5 D	N/A	1.05 +/- 0.075 D	1.13 +/- 0.056 D	1.29 +/- 0.039 D	1.45 +/- 0.159 D	1.60 +/- 0.055 D	1.05 +/- 0.024 D	1.05 +/- 0.097 D	1.32 +/- 0.137 D	1.49 +/- 0.140 D	1.53 +/- 0.151 D
36	331-350	AQIFFSLGPGFGVLLAFASY	18 D	N/A	0.45 +/- 0.033 D	0.46 +/- 0.088 D	0.52 +/- 0.141 D	0.35 +/- 0.146 D	0.47 +/- 0.136 D	0.52 +/- 0.130 D	0.57 +/- 0.156 D	0.39 +/- 0.038 D	0.39 +/- 0.114 D	0.32 +/- 0.107 D
37	340-349	GFGVLLAFAS	9 D	N/A	2.87 +/- 0.075 D	2.86 +/- 0.067 D	2.90 +/- 0.111 D	2.80 +/- 0.149 D	2.88 +/- 0.077 D	2.86 +/- 0.093 D	2.81 +/- 0.120 D	2.88 +/- 0.041 D	2.82 +/- 0.162 D	2.95 +/- 0.036 D
38	354-364	NNNCYQDALVT	10 D	N/A	0.19 +/- 0.079 D	0.19 +/- 0.054 D	0.10 +/- 0.025 D	0.09 +/- 0.187 D	0.16 +/- 0.084 D	0.18 +/- 0.068 D	0.14 +/- 0.073 D	0.07 +/- 0.050 D	0.13 +/- 0.189 D	0.01 +/- 0.109 D
39	354-366	NNNCYQDALVTSV	12 D	N/A	0.19 +/- 0.183 D	0.19 +/- 0.193 D	0.05 +/- 0.155 D	0.03 +/- 0.427 D	-0.07 +/- 0.271 D	0.22 +/- 0.128 D	0.17 +/- 0.167 D	-0.04 +/- 0.279 D	0.07 +/- 0.384 D	-0.13 +/- 0.350 D
40	383-398	LGYMAEMRNEDVSEVA	15 D	N/A	0.35 +/- 0.094 D	0.39 +/- 0.104 D	0.30 +/- 0.160 D	0.26 +/- 0.214 D	0.25 +/- 0.225 D	0.31 +/- 0.044 D	0.34 +/- 0.046 D	0.25 +/- 0.218 D	0.27 +/- 0.196 D	0.31 +/- 0.253 D
41	389-396	MRNEDVSE	7 D	N/A	0.31 +/- 0.089 D	0.35 +/- 0.102 D	0.20 +/- 0.014 D	0.29 +/- 0.150 D	0.34 +/- N/A D	0.30 +/- 0.055 D	0.34 +/- 0.059 D	0.27 +/- 0.084 D	0.32 +/- 0.105 D	0.26 +/- 0.056 D
42	397-406	VAKDAGPSLL	8 D	N/A	2.51 +/- 0.280 D	2.73 +/- 0.201 D	2.76 +/- 0.348 D	2.76 +/- 0.317 D	2.85 +/- 0.351 D	2.57 +/- 0.280 D	2.74 +/- 0.275 D	2.83 +/- 0.348 D	2.74 +/- 0.219 D	2.89 +/- 0.313 D
43	400-406	DAGPSLL	5 D	N/A	1.43 +/- 0.154 D	1.66 +/- 0.152 D	1.73 +/- 0.220 D	1.74 +/- 0.183 D	1.77 +/- 0.237 D	1.49 +/- 0.130 D	1.76 +/- 0.119 D	1.78 +/- 0.220 D	1.77 +/- 0.151 D	1.83 +/- 0.114 D
44	406-421	LFITYAEAIANMPAST	14 D	N/A	0.43 +/- 0.104 D	0.45 +/- 0.059 D	0.39 +/- 0.054 D	0.42 +/- 0.163 D	0.40 +/- 0.047 D	0.44 +/- 0.050 D	0.46 +/- 0.060 D	0.36 +/- 0.016 D	0.46 +/- 0.101 D	0.41 +/- 0.006 D
45	408-420	ITYAEAIANMPAS	11 D	N/A	0.21 +/- 0.160 D	0.19 +/- 0.154 D	0.18 +/- 0.194 D	0.21 +/- 0.171 D	0.12 +/- 0.196 D	0.18 +/- 0.117 D	0.20 +/- 0.163 D	0.17 +/- 0.015 D	0.20 +/- 0.148 D	0.16 +/- 0.086 D
46	408-422	ITYAEAIANMPASTF	13 D	N/A	0.43 +/- 0.050 D	0.41 +/- 0.048 D	0.34 +/- N/A D	0.35 +/- 0.147 D	0.35 +/- N/A D	0.42 +/- 0.002 D	0.43 +/- 0.026 D	0.31 +/- N/A D	0.37 +/- 0.074 D	0.34 +/- N/A D
47	483-499	FGGAYVVKLLEEYATGP	15 D	N/A	4.77 +/- 0.081 D	4.90 +/- 0.282 D	6.00 +/- 0.010 D	6.76 +/- 0.091 D	6.77 +/- 0.101 D	4.79 +/- 0.044 D	4.96 +/- 0.064 D	6.23 +/- 0.128 D	6.77 +/- 0.023 D	6.72 +/- 0.040 D
48	509-517	AVAVSWFYG	8 D	N/A	0.11 +/- 0.059 D	0.11 +/- 0.064 D	0.05 +/- 0.023 D	0.11 +/- 0.074 D	0.09 +/- 0.002 D	0.09 +/- 0.042 D	0.12 +/- 0.030 D	0.04 +/- 0.002 D	0.11 +/- 0.069 D	0.03 +/- 0.013 D
49	512-523	VSWFYGITQFCR	11 D	N/A	0.09 +/- N/A D	0.17 +/- N/A D	0.03 +/- N/A D	-0.22 +/- N/A D	0.02 +/- N/A D	0.05 +/- N/A D	0.11 +/- N/A D	-0.08 +/- N/A D	-0.20 +/- N/A D	-0.10 +/- N/A D
50	516-545	YGITQFCRDVKEMLGFSPGWFWRICWVAIS	28 D	N/A	0.88 +/- 0.076 D	0.91 +/- 0.091 D	0.79 +/- N/A D	0.77 +/- 0.333 D	0.88 +/- N/A D	0.87 +/- 0.040 D	0.87 +/- 0.094 D	0.66 +/- N/A D	0.83 +/- 0.169 D	0.77 +/- N/A D
51	519-548	TQFCRDVKEMLGFSPGWFWRICWVAISPLF	27 D	N/A	0.73 +/- 0.176 D	0.72 +/- 0.070 D	0.73 +/- N/A D	0.72 +/- 0.209 D	0.75 +/- N/A D	0.77 +/- 0.133 D	0.76 +/- 0.063 D	0.53 +/- N/A D	0.78 +/- 0.059 D	0.71 +/- N/A D
52	560-566	PPQLRLF	5 D	N/A	0.08 +/- 0.029 D	0.10 +/- 0.038 D	0.04 +/- 0.049 D	0.05 +/- 0.065 D	0.05 +/- 0.009 D	0.10 +/- 0.027 D	0.07 +/- 0.023 D	0.08 +/- 0.077 D	0.05 +/- 0.057 D	0.06 +/- 0.106 D
53	583-593	TSSFICIPTYI	9 D	N/A	0.17 +/- 0.060 D	0.18 +/- 0.042 D	0.15 +/- 0.148 D	0.11 +/- 0.219 D	0.11 +/- 0.169 D	0.19 +/- 0.063 D	0.17 +/- 0.033 D	0.12 +/- 0.129 D	0.11 +/- 0.146 D	0.09 +/- 0.147 D
54	590-595	PTYIAY	5 D	N/A	0.04 +/- 0.014 D	0.00 +/- 0.011 D	0.13 +/- 0.010 D	0.05 +/- 0.028 D	0.07 +/- 0.033 D	0.04 +/- 0.009 D	0.03 +/- 0.040 D	0.08 +/- 0.024 D	0.07 +/- 0.041 D	0.03 +/- 0.084 D
55	595-607	YRLIITPGTFKER	11 D	N/A	1.66 +/- 0.048 D	2.20 +/- 0.120 D	2.49 +/- 0.031 D	3.37 +/- 0.254 D	4.09 +/- 0.345 D	1.70 +/- 0.053 D	2.20 +/- 0.144 D	2.65 +/- 0.065 D	3.57 +/- 0.156 D	4.20 +/- 0.319 D
56	598-607	IITPGTFKER	8 D	N/A	1.54 +/- 0.281 D	1.99 +/- 0.103 D	2.37 +/- 0.429 D	3.06 +/- 0.341 D	3.79 +/- 0.351 D	1.59 +/- 0.243 D	2.07 +/- 0.194 D	2.48 +/- 0.452 D	3.28 +/- 0.356 D	3.77 +/- 0.381 D
57	601-606	PGTFKE	5 D	N/A	1.98 +/- 0.018 D	1.97 +/- 0.017 D	1.95 +/- 0.039 D	1.93 +/- 0.027 D	1.93 +/- 0.047 D	1.95 +/- 0.024 D	1.95 +/- 0.029 D	1.94 +/- 0.021 D	1.95 +/- 0.039 D	1.97 +/- 0.038 D
58	608-619	IIKSITPETPTE	9 D	N/A	2.47 +/- 0.240 D	2.65 +/- 0.211 D	3.85 +/- 0.241 D	4.68 +/- 0.181 D	4.77 +/- 0.151 D	2.51 +/- 0.175 D	2.69 +/- 0.273 D	4.13 +/- 0.294 D	4.68 +/- 0.152 D	4.81 +/- 0.113 D
59	616-634	TPTEIPCGDIRLNAVLVPR	15 D	N/A	7.92 +/- 0.140 D	8.00 +/- 0.055 D	8.01 +/- 0.248 D	7.87 +/- 0.229 D	7.99 +/- 0.225 D	7.90 +/- 0.207 D	8.00 +/- 0.071 D	8.01 +/- 0.202 D	7.86 +/- 0.233 D	8.00 +/- 0.259 D
60	623-641	GDIRLNAVLVPRGSHHHHH	17 D	N/A	8.04 +/- 0.155 D	8.14 +/- 0.122 D	8.16 +/- 0.219 D	8.15 +/- 0.211 D	8.27 +/- 0.324 D	8.03 +/- 0.168 D	8.08 +/- 0.189 D	8.21 +/- 0.319 D	8.15 +/- 0.203 D	8.20 +/- 0.600 D
61	625-629	IRLNA	4 D	N/A	1.93 +/- 0.146 D	1.94 +/- 0.131 D	1.88 +/- 0.245 D	1.85 +/- 0.240 D	1.87 +/- 0.257 D	1.89 +/- 0.148 D	1.89 +/- 0.164 D	1.87 +/- 0.273 D	1.88 +/- 0.194 D	1.84 +/- 0.292 D
62	631-643	LVPRGSHHHHHHH	11 D	N/A	1.67 +/- 1.524 D	1.95 +/- 1.502 D	4.44 +/- N/A D	3.91 +/- 1.088 D	5.38 +/- N/A D	1.62 +/- 1.641 D	1.93 +/- 1.508 D	4.43 +/- N/A D	3.87 +/- 1.110 D	5.38 +/- N/A D

Supplementary Table 4. HDX data summary table

**Data set**	Na^+ (reference)	K^+	5-HT	S-citalopram	Cocaine	Ibogaine
**HDX reaction details**	Buffer: (26 mM Tris (pH 8.0), 500 µM TCEP, 0.5 mM CHS, 24 µM of lipid mixture (POPC:POPE:POPG; 1:1:1 ratio), 1 mM DDM) containing either 300 mM NaCl or 300 mM KCl. Percent deuterium: 85%. Temperature: 25°C.					
**HDX time course**	0.25 min, 1 min, 10 min, 60 min, 480 min					
**HDX controls**	MX labeled control using 85% deuterium buffer (see above) and predigested hSERT					
**Back-exchange**	Na^+, K^+, 5-HT data set: Average – 50%; Maximum – 68%; Minimum – 26%.<br>Na^+, cocaine, ibogaine data set: Average – 50%; Maximum – 68%; Minimum – 25%.<br>Na^+, S-citalopram lacked a MX labeled sample for calculation of back exchange.					
**Number of peptides**	62 peptides					
**Sequence coverage**	69.1% of the sequence					
**Average peptide length / redundancy**	Average peptide length: 13.2 amino acids<br>The redundancy of each covered amino : 1.83					
**Replicates (biological or technical**	$n_{biological}$ = 1-3<br>$n_{technical}$ = 1-4	$n_{biological}$ = 1<br>$n_{technical}$ = 1-3	$n_{biological}$ = 1<br>$n_{technical}$ = 1-3	$n_{biological}$ = 3<br>$n_{technical}$ = 1-3	$n_{biological}$ = 2<br>$n_{technical}$ = 1-3	$n_{biological}$ = 2<br>$n_{technical}$ = 1-3
**Significant differences in HDX**	The difference in HDX had to be higher than the calculated 95% confidence interval for at least two measurement time points (see methods for additional criteria.					
**95% confidence interval**	Reference	0.34 D	0.35 D	0.43 D	0.45 D	0.45 D

Supplementary References

1. Merkle, P. S. *et al.* Substrate-modulated unwinding of transmembrane helices in the NSS transporter LeuT. *Sci. Adv.* **4**, eaar6179 (2018).
2. Weis, D. D., Engen, J. R. & Kass, I. J. Semi-automated data processing of hydrogen exchange mass spectra using HX-Express. *J. Am. Soc. Mass Spectrom.* **17**, 1700–1703 (2006).
3. Guttman, M., Weis, D. D., Engen, J. R. & Lee, K. K. Analysis of overlapped and noisy hydrogen/deuterium exchange mass spectra. *J. Am. Soc. Mass Spectrom.* **24**, 1906–12 (2013).
