# Supplementary material for: Conformational dynamics of the human serotonin transporter during substrate and drug binding
Source: Nat Commun. 2019 Apr 11;10:1687. doi: 10.1038/s41467-019-09675-z (PMC6459873; doi:10.1038/s41467-019-09675-z)
Supplement: Supplementary file 4 — Description of Additional Supplementary Files [file 41467_2019_9675_MOESM4_ESM.pdf]

Title: Supplementary dataset 1

Description: Overview of sequence information for the hSERT construct used in manuscript. Reference sequence is denoted ”U3854BA250-4.seq(1>1979)”. Quality control performed by the providing company. Reference sequence can be found in

Title: Supplementary dataset 2

Description: The sequencing data can be found in Supplementary dataset 3-5. Supplementary dataset 2 Overview of the hSERT construct used in manuscript

Title: Supplementary dataset 3

Description: Sequencing data for the hSERT construct used in manuscript. Quality control performed by the providing company

Title: Supplementary dataset 4

Description: Sequencing data for the hSERT construct used in manuscript. Quality control performed by the providing company

Title: Supplementary dataset 5

Description: Sequencing data for the hSERT construct used in manuscript. Quality control performed by the providing company

Title: Supplementary dataset 6

Description: HDX Data Tables and the HDX Data Summary Table according to the community-based recommendations
