# Supplementary material for: Conformational dynamics of the human serotonin transporter during substrate and drug binding
Source: Nat Commun. 2019 Apr 11;10:1687. doi: 10.1038/s41467-019-09675-z (PMC6459873; doi:10.1038/s41467-019-09675-z)
Supplement: Supplementary file 5 — Supplementary Data 1 [file 41467_2019_9675_MOESM5_ESM.pdf]

```
                                                        10        20        30        40        50        60        70        80
                                                  TCCACTTTGCCTTTCTCTCCACAGGTGTCCACTCCCAGTTCAATTACAGCTCTTAAGGGATCCCGGTCCGAAGCGCGCACGCG
A06-P66340-U3854BA250-4-U3854BA250-V-seqF.ab1(1>825)  →  TCCACTTTGCCTTTCTCTCCACAGGTGTCCACTCCCAGTTCAATTACAGCTCTTAAGGGATCCCGGTCCGAAGCGCGCACGCG
U3854BA250-4.seq(1>1979)                               →                                                                         GCGCGCACGCG

                                                        90       100       110       120       130       140       150       160
                                                  TGGAATTCGGCGCCATGGAGACGACGCCCTTGAATTCTCAGAAGCAGCTATCAGCGTGTGAAGATGGAGAAGATTGTCAGGAA
A06-P66340-U3854BA250-4-U3854BA250-V-seqF.ab1(1>825)  →  TGGAATTCGGCGCCaTGGAGACgACgCCCTTGAATTCTCAGAAGCAGCTATCAGCGTGTGAAGATGGAGAAGATTGTCAGGAA
U3854BA250-4.seq(1>1979)                               →  TGGAATTCGGCGCCATGGAGACGACGCCCTTGAATTCTCAGAAGCAGCTATCAGCGTGTGAAGATGGAGAAGATTGTCAGGAA

                                                       170       180       190       200       210       220       230       240
                                                  AACGGAGTTCTACAGAAGGTTGTTCCCACCCCAGGGGACAAAGTGGAGTCCGGGCAAATATCCAATGGGTACTCAGCAGTTCC
A06-P66340-U3854BA250-4-U3854BA250-V-seqF.ab1(1>825)  →  AACGGAGTTCTACAGAAGGTTGTTCCCACCCCAGGGGACAAAGTGGAGTCCGGGCAAATATCCAATGGGTACTCAGCAGTTCC
U3854BA250-4.seq(1>1979)                               →  AACGGAGTTCTACAGAAGGTTGTTCCCACCCCAGGGGACAAAGTGGAGTCCGGGCAAATATCCAATGGGTACTCAGCAGTTCC

                                                  250       260       270       280       290       300       310       320       330
                                                  AAGTCCTGGTGCGGGAGATGACACACGGCACTCTATCCCAGCGACCACCACCACCCTAGTGGCTGAGCTTCATCAAGGGGAAC
A06-P66340-U3854BA250-4-U3854BA250-V-seqF.ab1(1>825)  →  AAGTCCTGGTGCGGGAGATGACACACGGCACTCTATCCCAGCGACCACCACCACCCTAGTGGCTGAGCTTCATCAAGGGGAAC
U3854BA250-4.seq(1>1979)                               →  AAGTCCTGGTGCGGGAGATGACACACGGCACTCTATCCCAGCGACCACCACCACCCTAGTGGCTGAGCTTCATCAAGGGGAAC

                                                       340       350       360       370       380       390       400       410
                                                  GGGAGACCTGGGGCAAGAAGGTGGATTTCCTTCTCTCAGTGATTGGCTATGCTGTGGACCTGGGCAATGTCTGGCGCTTCCCC
A06-P66340-U3854BA250-4-U3854BA250-V-seqF.ab1(1>825)  →  GGGAGACCTGGGGCAAGAAGGTGGATTTCCTTCTCTCAGTGATTGGCTATGCTGTGGACCTGGGCAATGTCTGGCGCTTCCCC
U3854BA250-4.seq(1>1979)                               →  GGGAGACCTGGGGCAAGAAGGTGGATTTCCTTCTCTCAGTGATTGGCTATGCTGTGGACCTGGGCAATGTCTGGCGCTTCCCC

                                                     420       430       440       450       460       470       480       490
                                                  TACATATGTTACCAGAATGGAGGGGGGGCATTCCTCCTCCCCTACACCATCATGGCCATTTTTGGGGGAATCCCGCTCTTTTA
A06-P66340-U3854BA250-4-U3854BA250-V-seqF.ab1(1>825)  →  TACATATGTTACCAGAATGGAGGGGGGGCATTCCTCCTCCCCTACACCATCATGGCCATTTTTGGGGGAATCCCGCTCTTTTA
U3854BA250-4.seq(1>1979)                               →  TACATATGTTACCAGAATGGAGGGGGGGCATTCCTCCTCCCCTACACCATCATGGCCATTTTTGGGGGAATCCCGCTCTTTTA

                                                   500       510       520       530       540       550       560       570       580
                                                  CATGGAGCTCGCACTGGGACAGTACCACCGAAATGGATGCATTTCAATATGGAGGAAAATCTGCCCGATTTTCAAAGGGATTG
A06-P66340-U3854BA250-4-U3854BA250-V-seqF.ab1(1>825)  →  CATGGAGCTCGCACTGGGACAGTACCACCGAAATGGATGCATTTCAATATGGAGGAAAATCTGCCCGATTTTCAAAGGGATTG
U3854BA250-4.seq(1>1979)                               →  CATGGAGCTCGCACTGGGACAGTACCACCGAAATGGATGCATTTCAATATGGAGGAAAATCTGCCCGATTTTCAAAGGGATTG
```

```
                                                                  590       600       610       620       630       640       650       660
                                                          GTTATGCCATCTGCATCATTGCCTTTTACATTGCTTCCTACTACAACACCATCATGGCCTGGGCGCTATACTACCTCATCTCC
A06-P66340-U3854BA250-4-U3854BA250-V-seqF.ab1(1>825)  →  GTTATGCCATCTGCATCATTGCCTTTTACATTGCTTCCTACTACAACACCATCATGGCCTGGGCGCTATACTACCTCATCTCC
U3854BA250-4.seq(1>1979)                              →  GTTATGCCATCTGCATCATTGCCTTTTACATTGCTTCCTACTACAACACCATCATGGCCTGGGCGCTATACTACCTCATCTCC

                                                              670       680       690       700       710       720       730       740
                                                          TCCTTCACGGACCAGCTGCCCTGGACCAGCTGCAAGAACTCCTGGAACACTGGCAACTGCACCAATTACTTCTCCGAGGACAA
A06-P66340-U3854BA250-4-U3854BA250-V-seqF.ab1(1>825)  →  TCCTTCACGGACCAGCTGCCCTGGACCAGCTGCAAGAACTCCTGGAACACTGGCAACTGCACCAATTACTTCTCCGAGGACAA
U3854BA250-4.seq(1>1979)                              →  TCCTTCACGGACCAGCTGCCCTGGACCAGCTGCAAGAACTCCTGGAACACTGGCAACTGCACCAATTACTTCTCCGAGGACAA
A08-P66340-U3854BA250-4-U3854BA250-3-seq.ab1(1>829)   →                               CTGCaAGAACTCCTGGAACACTGGCAACTGCACCAATTACTTCTCCGAGGACAA

                                                           750       760       770       780       790       800       810       820       830
                                                          CATCACCTGGACCCTCCATTCCACGTCCCCTGCTGAAGAATTTTACACGCGCCACGTCCTGCAGATCCACCGATCGAAGGGGC
A06-P66340-U3854BA250-4-U3854BA250-V-seqF.ab1(1>825)  →  CATCACCTGGACCCTCCATTCCACGTCCCCTGCTGAAGAATTTTACACGCGCCACGTCCTGCAGATCCACCGATCGAA
U3854BA250-4.seq(1>1979)                              →  CATCACCTGGACCCTCCATTCCACGTCCCCTGCTGAAGAATTTTACACGCGCCACGTCCTGCAGATCCACCGATCGAAGGGGC
A08-P66340-U3854BA250-4-U3854BA250-3-seq.ab1(1>829)   →  CATCACCTGGACCCTCCATTCCACGTCCCCTGCTGAAGAATTTTACACGCGCCACGTCCTGCAGATCCACCGATCGAAGGGGC

                                                                   840       850       860       870       880       890       900       910
                                                          TCCAGGACCTGGGGGGCATCAGCTGGCAGCTGGCCCTCTGCATCATGCTGATCTTCACTGTTATCTACTTCAGCATCTGGAAA
U3854BA250-4.seq(1>1979)                              →  TCCAGGACCTGGGGGGCATCAGCTGGCAGCTGGCCCTCTGCATCATGCTGATCTTCACTGTTATCTACTTCAGCATCTGGAAA
A08-P66340-U3854BA250-4-U3854BA250-3-seq.ab1(1>829)   →  TCCAGGACCTGGGGGGCATCAGCTGGCAGCTGGCCCTCTGCATCATGCTGATCTTCACTGTTATCTACTTCAGCATCTGGAAA

                                                                920       930       940       950       960       970       980       990
                                                          GGCGTCAAGACCTCTGGCAAGGTGGTGTGGGTGACAGCCACCTTCCCTTATATCATCCTTTCTGTCCTGCTGGTGAGGGGTGC
U3854BA250-4.seq(1>1979)                              →  GGCGTCAAGACCTCTGGCAAGGTGGTGTGGGTGACAGCCACCTTCCCTTATATCATCCTTTCTGTCCTGCTGGTGAGGGGTGC
A08-P66340-U3854BA250-4-U3854BA250-3-seq.ab1(1>829)   →  GGCGTCAAGACCTCTGGCAAGGTGGTGTGGGTGACAGCCACCTTCCCTTATATCATCCTTTCTGTCCTGCTGGTGAGGGGTGC

                                                            1000      1010      1020      1030      1040      1050      1060      1070
                                                          CACCCTCCCTGGAGCCTGGAGGGGTGTTCTCTTCTACTTGAAACCCAACTGGCAAAAGCTTCTGGAGACAGGGGTGTGGATAG
U3854BA250-4.seq(1>1979)                              →  CACCCTCCCTGGAGCCTGGAGGGGTGTTCTCTTCTACTTGAAACCCAACTGGCAAAAGCTTCTGGAGACAGGGGTGTGGATAG
A08-P66340-U3854BA250-4-U3854BA250-3-seq.ab1(1>829)   →  CACCCTCCCTGGAGCCTGGAGGGGTGTTCTCTTCTACTTGAAACCCAACTGGCAAAAGCTTCTGGAGACAGGGGTGTGGATAG

                                                        1080      1090      1100      1110      1120      1130      1140      1150      1160
                                                          ATGCAGCCGCTCAGATCTTCTTCTCTCTTGGTCCGGGCTTTGGGGTCCTGCTGGCTTTTGCTAGCTACAACAAGTTCAACAAC
U3854BA250-4.seq(1>1979)                              →  ATGCAGCCGCTCAGATCTTCTTCTCTCTTGGTCCGGGCTTTGGGGTCCTGCTGGCTTTTGCTAGCTACAACAAGTTCAACAAC
A08-P66340-U3854BA250-4-U3854BA250-3-seq.ab1(1>829)   →  ATGCAGCCGCTCAGATCTTCTTCTCTCTTGGTCCGGGCTTTGGGGTCCTGCTGGCTTTTGCTAGCTACAACAAGTTCAACAAC
```

```
                                                  1170      1180      1190      1200      1210      1220      1230      1240
                                                  AACTGCTACCAAGATGCCCTGGTGACCAGCGTGGTGAACTGCATGACGAGCTTCGTTTCGGGATTTGTCATCTTCACAGTGCT
U3854BA250-4.seq(1>1979)                      →   AACTGCTACCAAGATGCCCTGGTGACCAGCGTGGTGAACTGCATGACGAGCTTCGTTTCGGGATTTGTCATCTTCACAGTGCT
A08-P66340-U3854BA250-4-U3854BA250-3-seq.ab1(1>829)  →   AACTGCTACCAAGATGCCCTGGTGACCAGCGTGGTGAACTGCATGACGAGCTTCGTTTCGGGATTTGTCATCTTCACAGTGCT

                                                  1250      1260      1270      1280      1290      1300      1310      1320
                                                  CGGTTACATGGCTGAGATGAGGAATGAAGATGTGTCTGAGGTGGCCAAAGACGCAGGTCCCAGCCTCCTCTTCATCACGTATG
U3854BA250-4.seq(1>1979)                      →   CGGTTACATGGCTGAGATGAGGAATGAAGATGTGTCTGAGGTGGCCAAAGACGCAGGTCCCAGCCTCCTCTTCATCACGTATG
A08-P66340-U3854BA250-4-U3854BA250-3-seq.ab1(1>829)  →   CGGTTACATGGCTGAGATGAGGAATGAAGATGTGTCTGAGGTGGCCAAAGACGCAGGTCCCAGCCTCCTCTTCATCACGTATG
A07-P66340-U3854BA250-4-U3854BA250-V-seqR.ab1(1>830) ←                                                       AGGTCCCAGCCTCCTCTTCATCACGTATG

                                                  1330      1340      1350      1360      1370      1380      1390      1400      1410
                                                  CAGAAGCGATAGCCAACATGCCAGCGTCCACTTTCTTTGCCATCATCTTCTTTCTGATGTTAATCACGCTGGGCTTGGACAGC
U3854BA250-4.seq(1>1979)                      →   CAGAAGCGATAGCCAACATGCCAGCGTCCACTTTCTTTGCCATCATCTTCTTTCTGATGTTAATCACGCTGGGCTTGGACAGC
A08-P66340-U3854BA250-4-U3854BA250-3-seq.ab1(1>829)  →   CAGAAGCGATAGCCAACATGCCAGCGTCCACTTTCTTTGCCATCATCTTCTTTCTGATGTTAATCACGCTGGGCTTGGACAGC
A07-P66340-U3854BA250-4-U3854BA250-V-seqR.ab1(1>830) ←   CAGAAGCGATAGCCAACATGCCAGCGTCCACTTTCTTTGCCATCATCTTCTTTCTGATGTTAATCACGCTGGGCTTGGACAGC

                                                  1420      1430      1440      1450      1460      1470      1480      1490
                                                  ACGTTTGCAGGCTTGGAGGGGGTGATCACGGCTGTGCTGGATGAGTTCCCACACGTCTGGGCCAAGCGCCGGGAGCGGTTCGT
U3854BA250-4.seq(1>1979)                      →   ACGTTTGCAGGCTTGGAGGGGGTGATCACGGCTGTGCTGGATGAGTTCCCACACGTCTGGGCCAAGCGCCGGGAGCGGTTCGT
A08-P66340-U3854BA250-4-U3854BA250-3-seq.ab1(1>829)  →   ACGTTTGCAGGCTTGGAGGGGGTGATCACGGCTGTGCTGGATGAGTTCCCACACGTCTGGGCCAAGCGCCGGGAGCGGTTCGT
A07-P66340-U3854BA250-4-U3854BA250-V-seqR.ab1(1>830) ←   ACGTTTGCAGGCTTGGAGGGGGTGATCACGGCTGTGCTGGATGAGTTCCCACACGTCTGGGCCAAGCGCCGGGAGCGGTTCGT

                                                  1500      1510      1520      1530      1540      1550      1560      1570
                                                  GCTCGCCGTGGTCATCACCTGCTTCTTTGGATCCCTGGTCACCCTGACTTTTGGAGGGGCCTACGTGGTGAAGCTGCTGGAGG
U3854BA250-4.seq(1>1979)                      →   GCTCGCCGTGGTCATCACCTGCTTCTTTGGATCCCTGGTCACCCTGACTTTTGGAGGGGCCTACGTGGTGAAGCTGCTGGAGG
A08-P66340-U3854BA250-4-U3854BA250-3-seq.ab1(1>829)  →   GCTCGCCGTGGTCATCACCTGCTTCTTT
A07-P66340-U3854BA250-4-U3854BA250-V-seqR.ab1(1>830) ←   GCTCGCCGTGGTCATCACCTGCTTCTTTGGATCCCTGGTCACCCTGACTTTTGGAGGGGCCTACGTGGTGAAGCTGCTGGAGG

                                                  1580      1590      1600      1610      1620      1630      1640      1650      1660
                                                  AGTATGCCACGGGGCCCGCAGTGCTCACTGTCGCGCTGATCGAAGCAGTCGCTGTGTCTTGGTTCTATGGCATCACTCAGTTC
U3854BA250-4.seq(1>1979)                      →   AGTATGCCACGGGGCCCGCAGTGCTCACTGTCGCGCTGATCGAAGCAGTCGCTGTGTCTTGGTTCTATGGCATCACTCAGTTC
A07-P66340-U3854BA250-4-U3854BA250-V-seqR.ab1(1>830) ←   AGTATGCCACGGGGCCCGCAGTGCTCACTGTCGCGCTGATCGAAGCAGTCGCTGTGTCTTGGTTCTATGGCATCACTCAGTTC

                                                  1670      1680      1690      1700      1710      1720      1730      1740
                                                  TGCAGGGACGTGAAGGAAATGCTCGGCTTCAGCCCGGGGTGGTTCTGGAGGATCTGCTGGGTGGCCATCAGCCCTCTGTTTCT
U3854BA250-4.seq(1>1979)                      →   TGCAGGGACGTGAAGGAAATGCTCGGCTTCAGCCCGGGGTGGTTCTGGAGGATCTGCTGGGTGGCCATCAGCCCTCTGTTTCT
A07-P66340-U3854BA250-4-U3854BA250-V-seqR.ab1(1>830) ←   TGCAGGGACGTGAAGGAAATGCTCGGCTTCAGCCCGGGGTGGTTCTGGAGGATCTGCTGGGTGGCCATCAGCCCTCTGTTTCT
```

```
                                                          1750      1760      1770      1780      1790      1800      1810      1820
                                                          CCTGTTCATCATTTGCAGTTTTCTGATGAGCCCGCCACAATTGCGACTTTTCCAATATAATTATCCTTACTGGAGTATCATCT
U3854BA250-4.seq(1>1979)                              →   CCTGTTCATCATTTGCAGTTTTCTGATGAGCCCGCCACAATTGCGACTTTTCCAATATAATTATCCTTACTGGAGTATCATCT
A07-P66340-U3854BA250-4-U3854BA250-V-seqR.ab1(1>830)  ←   CCTGTTCATCATTTGCAGTTTTCTGATGAGCCCGCCACAATTGCGACTTTTCCAATATAATTATCCTTACTGGAGTATCATCT

                                                          1830      1840      1850      1860      1870      1880      1890      1900
                                                          TGGGTTACTGCATAGGAACCTCATCTTTCATTTGCATCCCCACATATATAGCTTATCGGTTGATCATCACTCCAGGGACATTT
U3854BA250-4.seq(1>1979)                              →   TGGGTTACTGCATAGGAACCTCATCTTTCATTTGCATCCCCACATATATAGCTTATCGGTTGATCATCACTCCAGGGACATTT
A07-P66340-U3854BA250-4-U3854BA250-V-seqR.ab1(1>830)  ←   TGGGTTACTGCATAGGAACCTCATCTTTCATTTGCATCCCCACATATATAGCTTATCGGTTGATCATCACTCCAGGGACATTT

                                                          1910      1920      1930      1940      1950      1960      1970      1980      1990
                                                          AAAGAGCGTATTATTAAAAGTATTACCCCAGAAACACCAACAGAAATTCCTTGTGGGGACATCCGCTTGAATGCTGTGCTGGT
U3854BA250-4.seq(1>1979)                              →   AAAGAGCGTATTATTAAAAGTATTACCCCAGAAACACCAACAGAAATTCCTTGTGGGGACATCCGCTTGAATGCTGTGCTGGT
A07-P66340-U3854BA250-4-U3854BA250-V-seqR.ab1(1>830)  ←   AAAGAGCGTATTATTAAAAGTATTACCCCAGAAACACCAACAGAAATTCCTTGTGGGGACATCCGCTTGAATGCTGTGCTGGT

                                                          2000      2010      2020      2030      2040      2050      2060      2070
                                                          GCCGCGCGGCTCTCACCATCATCACCATCACCATCATCACCACCATCACTAAACCGGTGTCGACGCGGCCGCTCTAGAGCCTG
U3854BA250-4.seq(1>1979)                              →   GCCGCGCGGCTCTCACCATCATCACCATCACCATCATCACCACCATCACTAAACCGGT
A07-P66340-U3854BA250-4-U3854BA250-V-seqR.ab1(1>830)  ←   GCCGCGCGGCTCTCACCATCATCACCATCACCATCATCACCACCATCACTAAACCGGTGTCGACGCGGCCGCTCTAGAGCCTG

                                                          2080      2090      2100      2110      2120
                                                          CAGTCTCGACAAGCTTAATCAACCTCTGGATTACAAAATTTGtGAAAGATtGAC
A07-P66340-U3854BA250-4-U3854BA250-V-seqR.ab1(1>830)  ←   CAGTCTCGACAAGCTTAATCAACCTCTGGATTACAAAATTTGtGAAAGATtGAC
```
